# Cell–Surface Binding of DNA Nanostructures for Enhanced Intracellular and Intranuclear Delivery

**DOI:** 10.1021/acsami.3c18068

**Published:** 2024-03-18

**Authors:** Weitao Wang, Bhavya Chopra, Vismaya Walawalkar, Zijuan Liang, Rebekah Adams, Markus Deserno, Xi Ren, Rebecca E. Taylor

**Affiliations:** † Department of Mechanical Engineering, Carnegie Mellon University, Pittsburgh, Pennsylvania 15213, United States; ‡ Department of Physics, Carnegie Mellon University, Pittsburgh, Pennsylvania 15213, United States; § Department of Biomedical Engineering, Carnegie Mellon University, Pittsburgh, Pennsylvania 15213, United States; ∥ Department of Electrical and Computer Engineering, Carnegie Mellon University, Pittsburgh, Pennsylvania 15213, United States

**Keywords:** DNA nanostructure, DNA origami, cellular uptake, endocytosis, nuclear delivery, cell membrane engineering

## Abstract

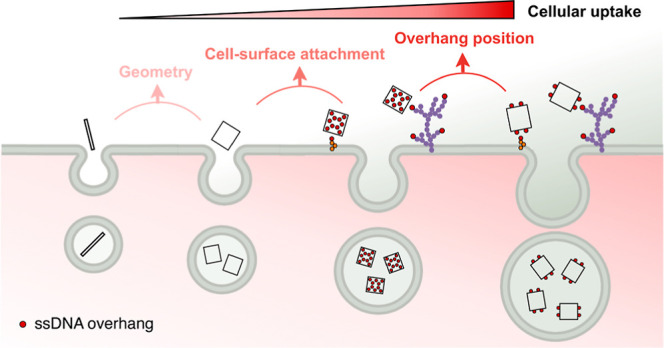

DNA nanostructures (DNs) have found increasing use in biosensing, drug delivery, and therapeutics because of their customizable assembly, size and shape control, and facile functionalization. However, their limited cellular uptake and nuclear delivery have hindered their effectiveness in these applications. Here, we demonstrate the potential of applying cell-surface binding as a general strategy to enable rapid enhancement of intracellular and intranuclear delivery of DNs. By targeting the plasma membrane via cholesterol anchors or the cell-surface glycocalyx using click chemistry, we observe a significant 2 to 8-fold increase in the cellular uptake of three distinct types of DNs that include nanospheres, nanorods, and nanotiles, within a short time frame of half an hour. Several factors are found to play a critical role in modulating the uptake of DNs, including their geometries, the valency, positioning and spacing of binding moieties. Briefly, nanospheres are universally preferable for cell surface attachment and internalization. However, edge-decorated nanotiles compensate for their geometry deficiency and outperform nanospheres in both categories. In addition, we confirm the short-term structural stability of DNs by incubating them with cell medium and cell lysate. Further, we investigate the endocytic pathway of cell-surface bound DNs and reveal that it is an interdependent process involving multiple pathways, similar to those of unmodified DNs. Finally, we demonstrate that cell-surface attached DNs exhibit a substantial enhancement in the intranuclear delivery. Our findings present an application that leverages cell-surface binding to potentially overcome the limitations of low cellular uptake, which may strengthen and expand the toolbox for effective cellular and nuclear delivery of DNA nanostructure systems.

## Introduction

Nanoscale platforms have drawn enormous research effort in the past decade for imaging, biosensing, and drug delivery.^1^ These nanoplatforms can carry molecular loads, including diagnostic and therapeutic agents, serving as effective delivery vehicles to target specific cell types and intracellular compartments. Nevertheless, their internalization and accumulation inside of cells have raised concerns over cytotoxicity.^2^ Moreover, the need for personalized therapeutics and diagnosis requires versatile and programmable delivery systems, which limits our choice of delivery vehicles.^3^

Structural DNA nanotechnology is recognized as a practicable candidate to potentially overcome the aforementioned obstacles due to its biocompatibility, biodegradability, programmability, and ease of functionalization.^4−7^ Since its emergence, myriad DNA nanostructures (DNs) have been designed, synthesized with nanoscale precision, and modified with chemical and biological conjugates for biomedical applications.^7−10^ For example, recent studies show the potential of DNs as genetic cargo for gene expression and therapeutics.^11−14^ Despite the achievements made, one challenge that remains is the cellular uptake of DNs, which is limited in both the amount and the speed.^15^ Previously, this topic has been studied in terms of geometric parameters like size and shape, wireframe architecture, molecular weight, and surface chemistry.^16−19^ For instance, Bastings et al. reported that compact and solid DNs with low aspect ratios resulted in substantially higher DN uptake after overnight incubation.^16^ Moreover, DNs could trigger cellular uptake via interactions with specific membrane receptors.^15^ This has sparked further studies, such as programming DNs to bind to membrane receptors and activate endocytosis, and decorating DNs with ligands, such as aptamers, peptides, and proteins to enhance DN uptake.^20−22^ However, taking these factors into consideration, the amount of internalized DNs may still be insufficient to fulfill their therapeutic capabilities. Moreover, many studies require a lengthy incubation period, typically lasting a couple of hours or even 24 h, to achieve improved internalization.^16,17,19^ This slow speed of cellular uptake presents an additional challenge.

Applying theoretical analysis and molecular dynamics simulations, previous studies have demonstrated that reducing the separation between substances and cellular membranes triggered stronger membrane–substance interaction, which facilitated the internalization.^23,24^ A more recent experimental study has suggested that a stronger membrane-DN interaction may lead to preferable DN uptake.^17^ These results raise the question of whether DN uptake can be enhanced by simply bringing DNs and cellular membranes into close proximity without harnessing the additional peptides or ligands. Only a limited number of studies have explored this topic and provided support for the hypothesis. For example, 6-duplex nanobundles modified with cholesterol anchors had significant enhancement in their uptake as compared to unmodified nanobundles.^25^ Binding DNA nanotiles onto the cell-surface glycocalyx also enhanced nanotile’s uptake.^26^ While these studies provide new insights into the relation between an enhanced membrane-DN interaction and an increase in NP uptake, the topic has not been fully explored, and many important questions need to be answered, for instance, if this relation applies to a broader range of DNs, and how to engineer and optimize such an enhanced interaction. Further, it is unclear if different endocytic pathways will be utilized.

Toward this end, here we performed a comprehensive study with the aim of understanding the relation between cell-surface binding of DNs and their cellular uptake. We first revisited how DN internalization was affected by its geometry using DNA nanospheres, nanorods, and nanotiles. By modifying DNs with membrane binding moieties, cholesterol-mediated membrane anchoring was performed using both a two-step and recently developed one-step method while glycocalyx anchoring was performed through hybridizing to click-anchored ssDNA. We further explored the impact of binding moiety valency, placement, and spacing on DN uptake and found that all factors played significant roles in modulating uptake. We also assessed the structural stability of DNs by incubating them with both cell medium and cell lysate. Lastly, we examined the endocytic pathways of cell-surface bound DNs and investigated their intranuclear delivery.

## Results and Discussion

### Design, Synthesis, and Characterization of DNs

**Figure 1:**
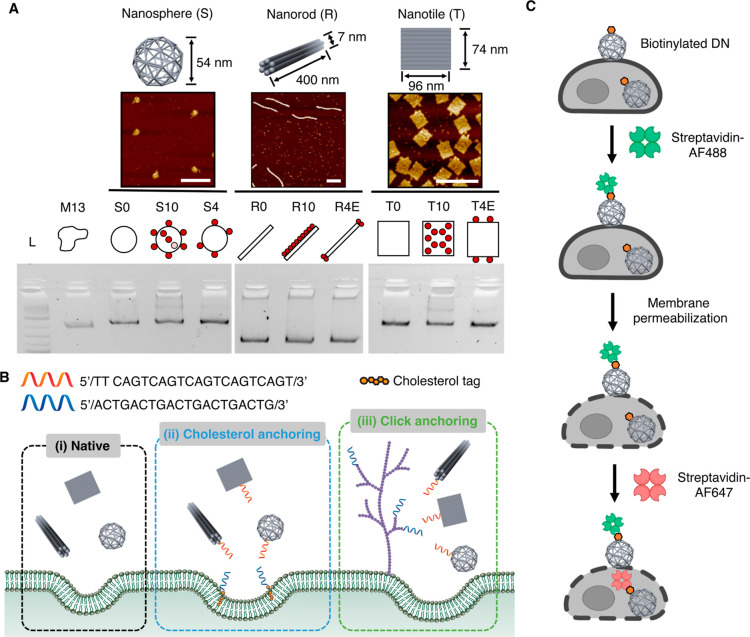
An overview of the design and characterization of DNs, cell-surface binding strategies, and dual-color fluorescence staining. (A) AFM and agarose gel electrophoresis characterizations of purified DNA nanospheres (S), nanorods (R), and nanotiles (T). All scale bars: 200 nm. Red dots represent the ssDNA overhangs. (B) Three cell-surface binding strategies, including (i) native adhesion for unmodified DNs without overhangs, (ii) two-step cholesterol membrane anchoring, and (iii) two-step click glycocalyx anchoring. (C) Dual-color fluorescence staining. After cell fixation, cell-surface attached DNs were first stained with streptavidin-Alexa Fluor 488. Cellular plasma membranes were then permeabilized using Triton X-100. Lastly, streptavidin-Alexa Fluor 647 was administrated to stain the internalized DNs.

The shape of the nanoparticles affects their interactions with cells, including their binding affinity to cell membranes and internalization efficiency.^16,27^ In this study, three shapes of DNs, including DNA nanospheres (*S*, ∼54 nm in diameter), nanorods (*R*, ∼7 nm in diameter and ∼400 nm in length), and nanotiles (*T*, 96 × 74 nm), were designed and synthesized (Figure1, Tables S1–S4). All DNs were labeled with 14 biotin molecules for subsequent fluorescence staining. In order to investigate the effect of the valency and placement of single-stranded DNA (ssDNA) overhang on cellular uptake, each shape of DNs was modified with three types of ssDNA overhang decorations: 0 overhang (S0, R0, T0), 10 overhangs uniformly presented on the central surface (S10, R10, T10), and 4 overhangs with 2 overhangs on two edges of the DN (S4, R4E, T4E. Since a sphere does not differ at center and edge on its surface, we used S4 instead of S4E). All DNs were characterized with atomic force microscopy (AFM) and agarose gel electrophoresis, which confirmed their formation as well as high yields (Figure S2).

### Cell-Surface Binding-Enhanced Cellular Uptake of DNs

Human umbilical vein endothelial cells (HUVECs) play a critical role in maintaining vascular homeostasis and are involved in numerous physiological and pathological processes, including angiogenesis, inflammation, thrombosis, as well as endothelial dysfunction and related vascular diseases such as atherosclerosis and hypertension.^28^ Studying the cellular uptake of HUVECs may provide valuable insights for the development of therapeutics related to the vascular system. In this study, they serve as a model example.

Three strategies were studied to target DNs onto cell surface (Figure 1B). Bare DNs can attach to cell membranes through a combination of intrinsic native interactions, including electrostatic forces and binding to scavenger receptors like LOX-1.^29^ This condition is commonly employed in the majority of DN cellular uptake investigations, referred to as native adhesion, and serves as a negative control in this study.^16−18^ For overhang-decorated DNs that can target cell membranes, a two-step cholesterol membrane anchoring and two-step click glycocalyx anchoring were used.^26,30^ In cholesterol anchoring, cell membranes were first immobilized with cholesterol-ssDNA anchors by spontaneously inserting the hydrophobic portion of the amphiphile into the lipid membranes. In a second step, DNs modified with the complementary ssDNA hybridized with the membrane-immobilized ssDNA anchors, leading to the recruitment of DNs onto the membrane. For click glycocalyx anchoring, ssDNA anchors were incorporated onto cell-surface glycocalyx through metabolic glycan labeling and copper-free click chemistry.^26^ Then DNs carrying complementary ssDNA hybridized with glycocalyx-immobilized ssDNA anchors, attaching themselves onto the glycocalyx.

In order to simultaneously visualize and quantify both the cell-surface DNs and internalized DNs, we utilized a dual-color fluorescence staining method (Figure 1C).^26^ Cells were first fixed after incubating with biotinylated DNs. Next, streptavidin-Alexa Fluor 488 (AF488) was introduced to stain and saturate the biotin molecules on cell-surface DNs, followed by the permeabilization of cellular plasma membranes. Internalized DNs were then stained by administering streptavidin-Alexa Fluor 647 (AF647). The administration of streptavidin-AF488 to prepermeabilized cells did not result in a noticeable increase in the intracellular signal (Figure S3). Fluorescence microscopy images were taken to assess the cell surface binding and internalization of DNs. For quantitative measurement, we utilized a previously reported computational method for facile assessment.^26,31^ In order to capture the fluorescence signals from the internalized DNs from cells across the *z* direction, we utilized epifluorescence microscopy for imaging. Our computational pipeline greatly reduces undesired background noise and more accurately captures the signals of internalized DNs, which lowers the impact from autofluorescence and the noise caused by nonspecific internalization of fluorophores (Figure S4A). Flow cytometry data confirms the feasibility and validity of this method (Figure S4B). It is important to note that the fluorophores used for staining the cell-surface and internalized DNs have different excitation and emission wavelengths. As a result, direct comparison of their fluorescence intensities is not meaningful. Hence, we will limit our comparisons to within each respective category.

To answer the question of whether cell-surface binding enhances the cellular uptake of DNs, we administered 9 DNs to HUVECs, respectively, and incubated for 30 min at 37 °C. The cell-surface and internalization signals were measured, and an internalization efficiency was defined by dividing internalization signal intensity by the sum of internalization and cell-surface signal intensities (eq 1).
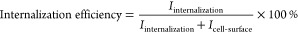
1

**Figure 2:**
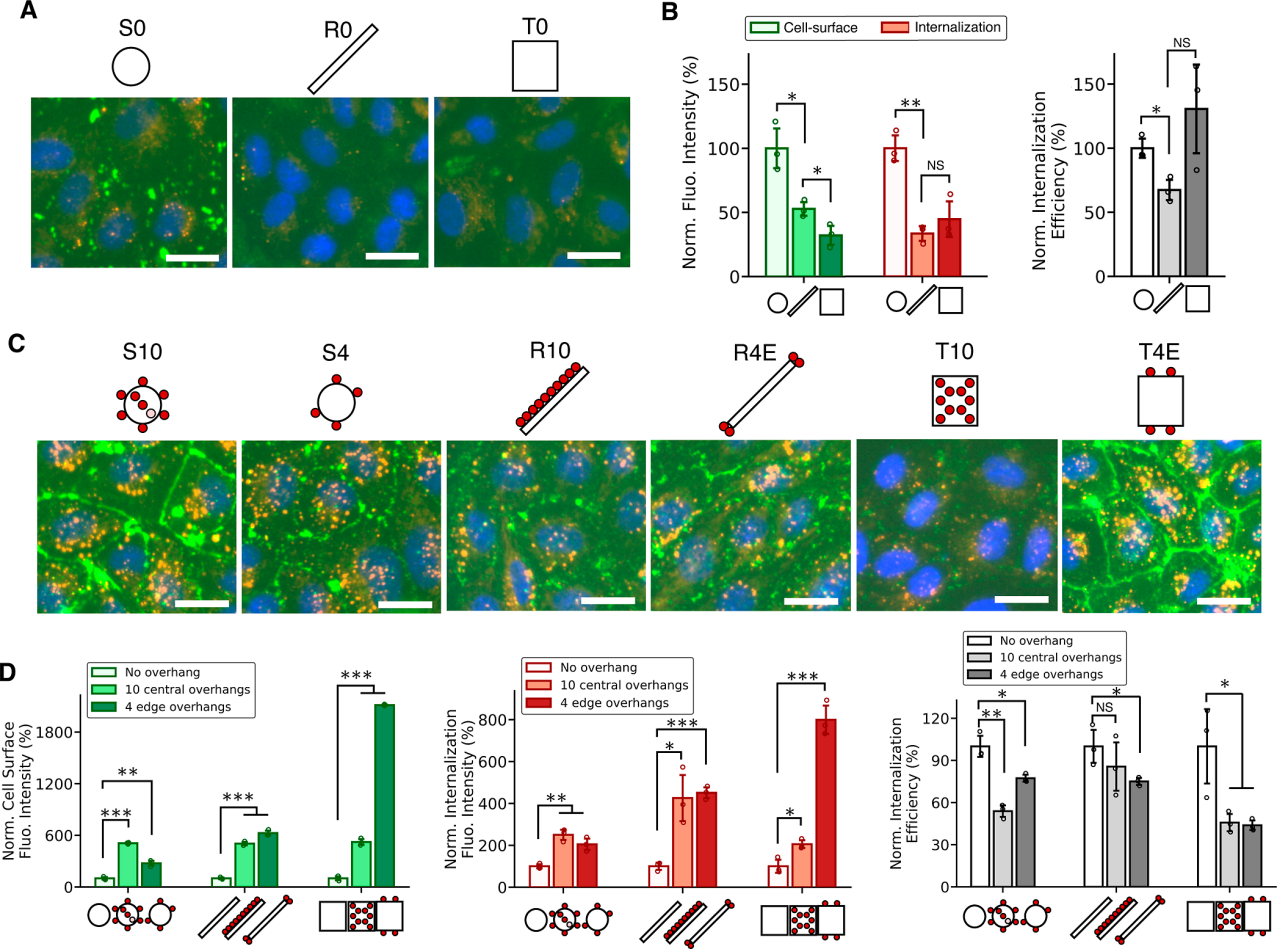
Cell-surface binding and internalization of cell-surface attached DNs via cholesterol membrane anchoring. (A) Fluorescence microscopy images of HUVECs after incubating with DNs with no overhangs for 30 min at 37 °C. (B) Quantification of fluorescence intensities of cell-surface and internalization signals, and internalization efficiency of DNs with no overhangs. In all three categories, the data were normalized to the readouts of nanospheres. (C) Fluorescence microscopy images of HUVECs after incubating with DNs with overhangs for 30 min at 37 °C. (D) Quantification of the fluorescence intensities of cell-surface signal, internalization signal and internalization efficiency of DNs with overhangs. In each shape, the data were normalized to DNs with no overhangs. Briefly, S10 and S4, R10 and R4E, T10 and T4E were normalized to S0, R0, and T0, respectively. For all fluorescence microscopy images, cell-surface DNs were stained with streptavidin-AF488 (green) and internalized DNs were stained with streptavidin-AF647 (red). Cell nuclei were stained with Hoechst (blue). The absolute values of fluorescence signal intensities are provided in Figure S6. Data were presented as means ± s.d. in (B) and (D) with *n* = 3. *P ≤ 0.05, **P ≤ 0.01, ***P ≤ 0.001. All scale bars: 25 μm.

The efficiency metric was intended to take cell-surface DNs into account in a consistent manner across all conditions, without indicating the actual fraction of internalized DNs. We first revisited the geometry effect by comparing the native membrane adhesion and internalization of DNs with no overhangs. We found that nanospheres outperformed nanorods and nanotiles in both cell-surface and internalization signal intensities, agreeing with previous studies (Figure 2A,B).^16,17^ Surprisingly, though having low signal intensities, nanotiles possessed a higher internalization efficiency as compared to two other shapes. To study the effect of overhangs on cell-surface binding and internalization, the readouts of DNs with overhangs were normalized to unmodified DNs in each shape. For instance, S10 and S4 were normalized to S0 in the cell-surface, internalization, and efficiency plot. As shown in Figure 2C,D, all three shapes of DNs exhibited significant enhancement in their membrane binding (at least 3-fold and up to 20-fold) and internalization (at least 2-fold and up to 8-fold) via cholesterol anchoring after just 0.5 h incubation. The rapid internalization process substantially reduces the need for long incubation periods which have been reported by prior studies that several hours to up to 24 h of incubation were needed to achieve improved cellular uptake.^16,17,32^ The potential effect of cell membrane modification with cholesterol on DN internalization appeared negligible (Figure S5). The shape of DNs and the placement of overhangs, however, greatly impacted not only cell-surface binding of the DNs but also their internalization. Nanospheres were in general more preferentially attached to the cell surface and internalized than nanorods and nanotiles. However, edge-decorated nanotiles T4E outperformed all other DNs with only 4 overhangs (Figure S6), which can be attributed to the previously reported sharp nanostructural features (ends, edges, corners) facilitating the membrane interactions with DNs.^30,33^ Surprisingly, with the enhancement in the uptake of DNs with overhangs, their internalization efficiency dropped compared to bare DNs with no overhangs. The trend held for all shapes, presumably because the cellular uptake efficiency had plateaued while DNs in the buffer continued to bind to membranes. In addition, the cell viability after incubation with DNs was examined and no significant difference was observed compared to pretreated cells (Figure S7). These findings support our assumption that using a cholesterol anchoring strategy to bind DNs onto cell plasma membranes, we were able to achieve rapid enhancement of the cellular uptake of DNs.

**Figure 3:**
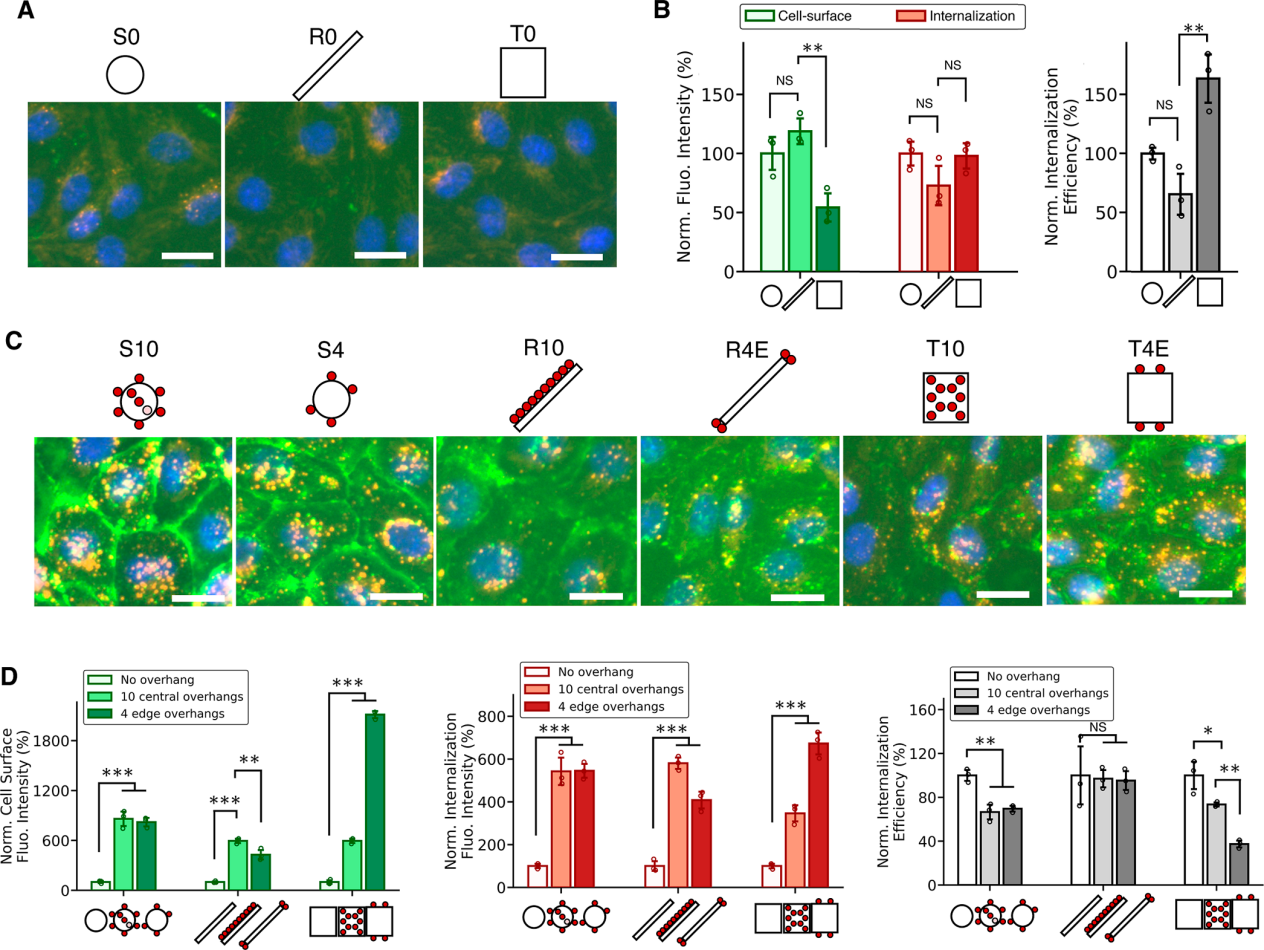
Cell-surface binding and internalization of cell-surface attached DNs via two-step click glycocalyx anchoring. (A) Fluorescence microscopy images of HUVECs after incubating with DNs with no overhangs for 30 min at 37 °C. (B) Quantification of fluorescence intensities of cell-surface and internalization signals, and internalization efficiency of DNs with no overhangs. In all three categories, the data were normalized to the readouts of nanospheres. (C) Fluorescence microscopy images of HUVECs after incubating with DNs with overhangs for 30 min at 37 °C. (D) Quantification of the fluorescence intensities of cell-surface signal, internalization signal, and internalization efficiency of DNs with overhangs. In each shape, the data were normalized to DNs with no overhangs. For all fluorescence microscopy images, cell-surface DNs were stained with streptavidin-AF488 (green) and internalized DNs were stained with streptavidin-AF647 (red). Cell nuclei were stained with Hoechst (blue). The absolute values of fluorescence signal intensities are provided in Figure S8. Data were presented as means ± s.d. in (B) and (D) with *n* = 3. *P ≤ 0.05, **P ≤ 0.01, ***P ≤ 0.001. All scale bars: 25 μm.

To investigate if other cell-surface binding strategies also lead to an enhancement in DN uptake, we turned to click glycocalyx anchoring in which DNs were anchored onto cell-surface glycocalyx. After 0.5 h incubation, the native adhesion and internalization of DNs with no overhangs seemed to be lower compared to the control groups with cholesterol anchoring, potentially due to the modification of glycocalyx (Figure 3A,B). Both cell-surface and internalization signals with overhangs increased similarly in click anchoring as in cholesterol anchoring (Figure 3C,D). The importance of the valency and position of overhangs was demonstrated again, especially when comparing nanorods R4E and R10, as well as nanotiles T4E and T10. Notably, T4E again had the most internalization among all structures (Figure S8). To briefly summarize, we observed a consistent relation between an enhancement in cell-surface binding of DNs and a similar increase in internalization of DNs, in both cholesterol membrane anchoring and click glycocalyx anchoring, showcasing the general appliance of the strategy that utilizes cell-surface binding for rapid cellular uptake enhancement.

**Figure 4:**
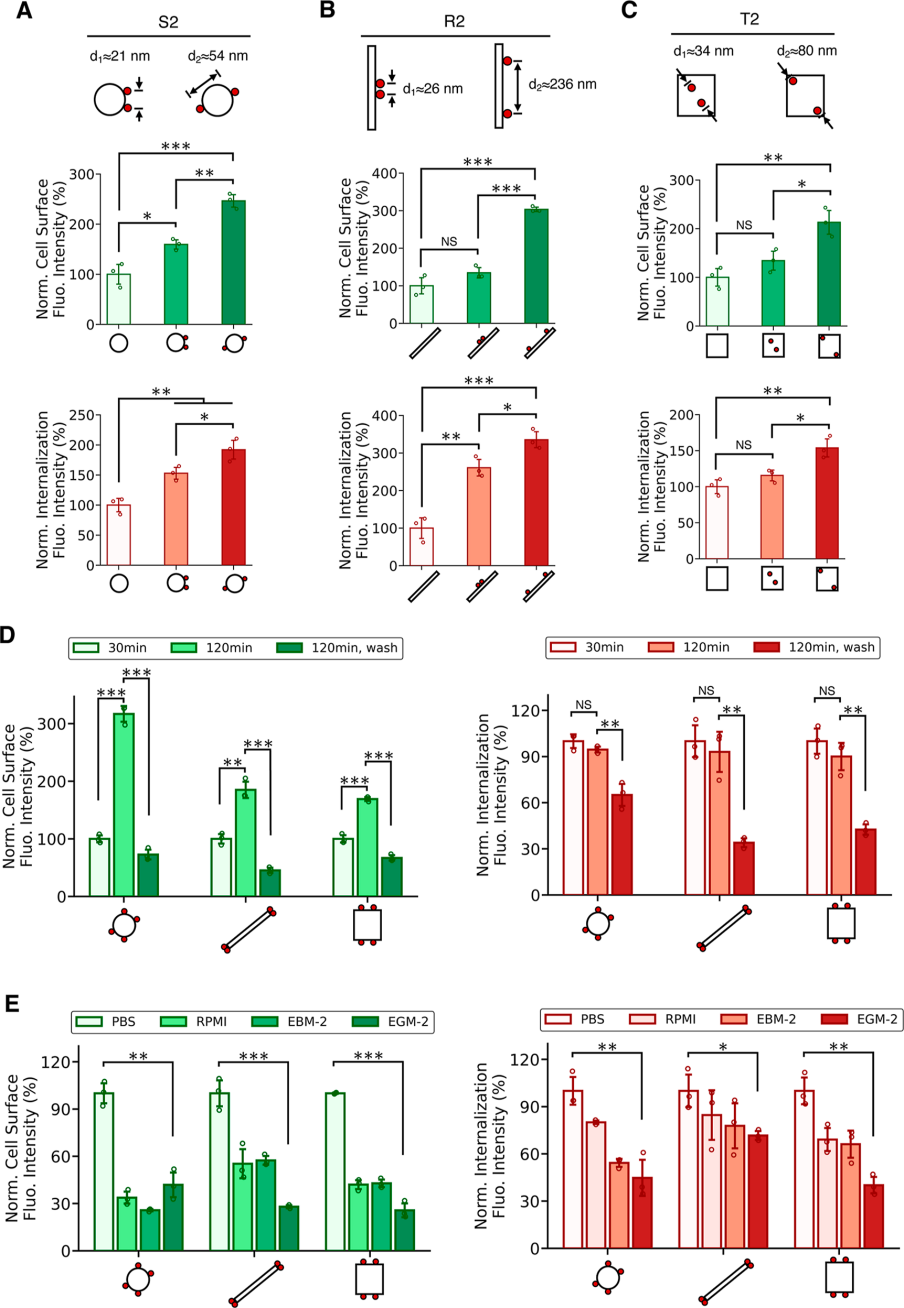
Effects of overhang spacing, incubation time, and buffer on the cell-surface binding and internalization of DNs using cholesterol anchoring. Membrane binding and cell internalization of DNs decorated with 2 overhangs with two spacing distances. For (A) DNA spheres, *d*_1_ ≈ 21 and *d*_2_ ≈ 54 nm; for (B) rods, *d*_1_ ≈ 26 and *d*_2_ ≈ 236 nm; for (C) tiles, *d*_1_ ≈ 34 and *d*_2_ ≈ 80 nm. In (A)–(C), cells were incubated with DNs for 30 min. (D) Membrane binding and cell internalization of DNs under three incubation time scales: 30 min incubation, 120 min incubation, and a two-step 120 min incubation: 30 min initial incubation followed by washing and 90 min subsequent incubation. (E) Membrane binding and cell internalization of DNs under four buffer conditions: PBS, RPMI 1640, EBM-2, and EGM-2. Data were presented as means ± s.d. with *n* = 3. *P ≤ 0.05, **P ≤ 0.01, ***P ≤ 0.001.

We further investigated other factors that might affect the uptake of DNs, including overhang spacing, incubation time, the concentration of DNs and cholesterol anchors, and the incubation buffer. First, the overhang spacing may affect the interaction between DNs and membrane anchors.^30^ We changed the spatial placement of two overhangs on three shapes of DNs. The distance between overhangs was adjusted to around 21 and 54 nm for spheres, 26 and 236 nm for rods, and 34 and 80 nm for tiles (Figures 4A–C and S1). We found that in all shapes, an increasing spacing between overhangs resulted in an enhancement in the membrane binding and internalization of DNs using cholesterol anchoring method. This observation may again be attributed to the hypothesis that overhangs positioned closer to the edge of DNs can more efficiently assess the membrane anchors, thereby facilitating the binding and cellular uptake.^30,33^

Next, we employed different incubation conditions. We noticed that while cell-surface-bound DNs continued to increase with the extended 2 h incubation, the internalization signal appeared to plateau, with no significant changes for all three edged-decorated shapes (Figure 4D). However, if the cells were first incubated with DNs, then washed and followed by another round of incubation, we saw a rapid decrease in cell-surface and internalization signals. We reason that after washing, there were no DNs in the buffer available to continue binding to cell membranes, thus limiting the membrane binding internalization of DNs.

The concentration of DNs and cholesterol anchors positively affected the membrane binding and uptake of DNs, as reported previously.^16,18^ By adjusting DN concentration from 1, 5, to 10 nM, or the concentration of cholesterol anchors administrated to cells, from 0.1, 0.5, to 1 μM, we observed a monotonic increase in both the cell-surface and internalization signal intensities with an increasing concentration of DNs or cholesterol anchors (Figures S9 and S10). However, it is important to notice that an excessively high concentration of cholesterol anchors led to significant aggregation of DNs on cell membranes (Figure S10).

DNA origami rely on the positive ions in the buffer for structure formation and maintenance.^4−6^ This leads to the question of whether the buffer has an influence on the membrane binding and uptake of DNs. Here we used four buffers, including phosphate buffered saline (PBS), Roswell Park memorial institute (RPMI) 1640 medium, endothelial cell growth basal medium-2 (EBM-2), and endothelial cell growth medium-2 (EGM-2). We found DNs in PBS buffer were most preferably bound to cell membranes and internalized than other cell medium (Figure 4E). DNs in EGM-2 were the least preferable. The buffer may affect the result from various aspects. For instance, the ions and fetal bovine serum (FBS) in the buffer have an influence on the stability of DNs.^34−36^ Both the type and concentration of ions could affect how DNs interact with the cell surface and subsequently bind to the membrane.^37,38^ Furthermore, ions might also modulate cellular activities, including membrane tension, membrane movement, and cellular uptake.^39,40^ Thus, in this study, we only compare results of cells incubated with the same buffer, PBS in most cases, unless otherwise noted.

### One-step Cholesterol Anchoring versus Two-step Cholesterol Anchoring

Lipophilic cholesterol tags have been widely used as a modification to DNs, enabling DNs to interact with and bind to cellular membranes.^41−43^ Yet the modification with hydrophobic cholesterol tags on DNs may lead to undesired aggregation of DNs, damaging their structural monodispersity and thus limiting the applications.^44,45^ It is for this reason, the two-step cholesterol anchoring method was developed to avoid the aggregation problem.^7,26,30^ Recently, Ohmann et al. have demonstrated that a strategic choice of cholesterol-conjugated sequences can successfully control the aggregation of cholesterol-modified DNs.^44,45^ To place cholesterol TEG linkers on an overhang sequence, incorporating an ssDNA overhang adjacent to the cholesterol and thus presenting a shielding effect is able to control the aggregation and increase the yield of one-pot cholesterol-conjugated DNs. Studies on lipid vesicles, such as GUVs or SUVs, have shown that the spontaneous insertion of cholesterol into lipid membranes was not impeded by the presence of a shielding overhang in the one-pot assembly of cholesterol-conjugated DNs.^44,45^ The successful synthesis of cholesterol-conjugated DNs opens new possibilities for in vivo research where two-step methods are unpractical. Therefore, we think it is important to investigate the difference in the membrane binding and cellular uptake between DNs with direct conjugation of cholesterol tags, which we refer to as the one-step cholesterol anchoring, and the two-step cholesterol anchoring, to provide guidance in the future applications of cholesterol-modified DNs.

**Figure 5:**
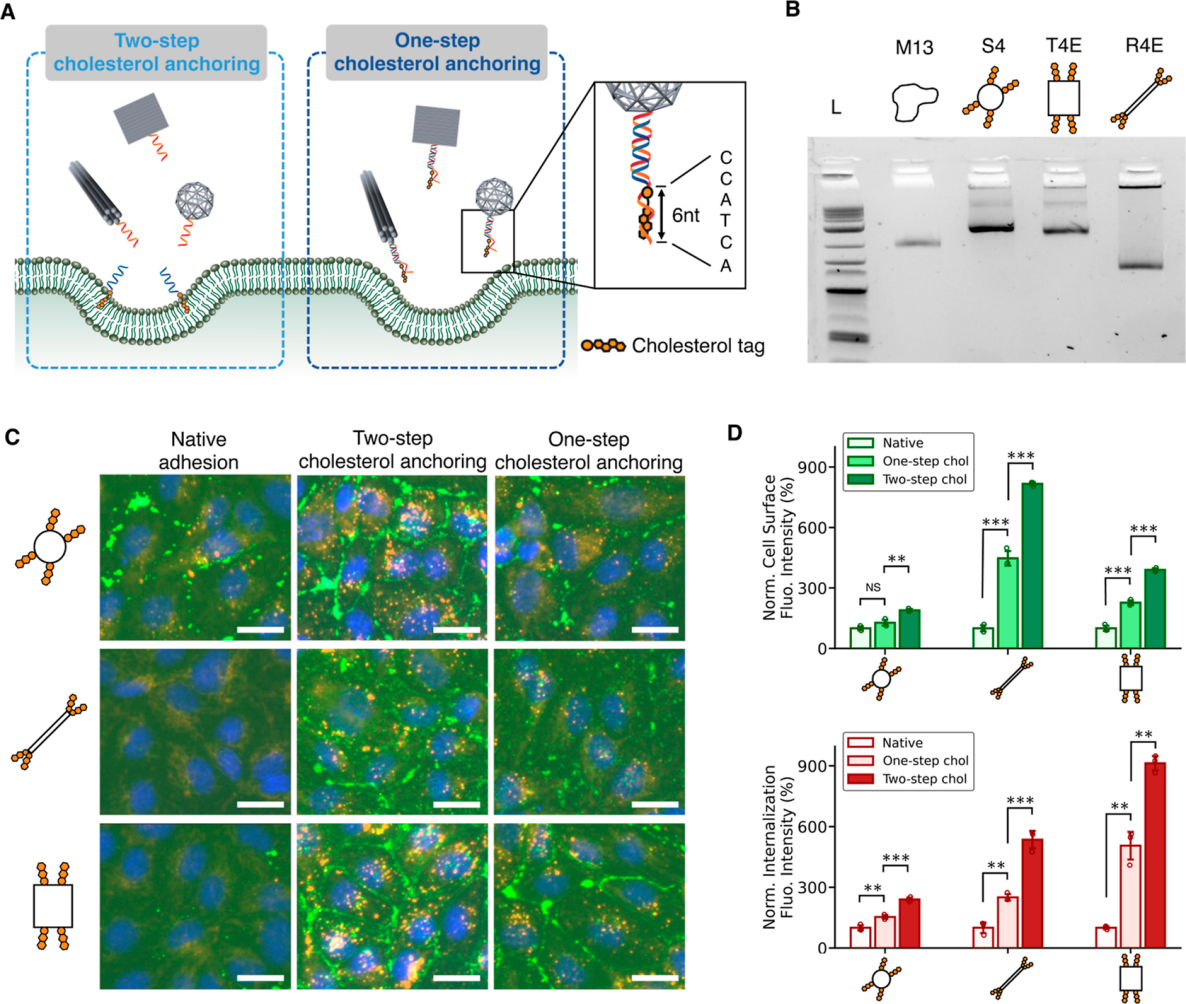
A comparison between one-step cholesterol anchoring and two-step cholesterol anchoring on the membrane binding and cellular uptake of DNs. (A) Schematic illustrations of one-step cholesterol anchoring. Cholesterol molecules were directly conjugated onto DNs with a shielding ssDNA overhang adjacent to the cholesterol. (B) Agarose gel electrophoresis of DNA nanospheres, nanorods, and nanotiles conjugated with four cholesterol molecules on their edges. (C) Fluorescence microscopy images of HUVECs after incubating with DNs for 30 min using one-step and two-step cholesterol anchoring. Membrane-bound DNs were stained with streptavidin-AF488 (green) and internalized DNs were stained with streptavidin-AF647 (red). Cell nuclei were stained with Hoechst (blue). Scale bars: 25 μm. (D) Quantification of fluorescence intensities of cell-surface and internalization signals for DNs using one-step and two-step cholesterol anchoring. The data were normalized to unmodified DNs. Data were presented as means ± s.d. with *n* = 3. **P ≤ 0.01, ***P ≤ 0.001.

For this one-step approach, we modified nanosphere S4, nanorod R4E and nanotile T4E by directly conjugating cholesterol tags onto the edges and incorporating an additional complementary ssDNA for a shielding effect (Figure 5A). The complementary ssDNA is 6-base longer than the cholesterol-conjugated sequence. Monodispersed bands were observed for all DNs in agarose gel electrophoresis with no significant aggregations, confirming the successful formation of DNs (Figure 5B). Fluorescence microscopy images of HUVECs showed the preferential membrane binding and uptake of DNs with one-step cholesterol anchoring compared to unmodified DNs (Figure 5C). Nanospheres outperformed nanotiles and nanorods, demonstrating the geometry effect still held for the one-step approach. Intriguingly, compared to two-step cholesterol anchoring, the cell-surface and internalization signal intensities of one-step cholesterol anchoring were significantly weaker (Figure 5D). Considering that DNs in both methods had the same number of tags or overhangs, this finding suggests that the efficiency of the spontaneous insertion of cholesterol into lipid membranes might be lower if the cholesterol is directly conjugated on DNs. Nonetheless, the highlevel of cell-surface and internalization signals validate the one-step approach as a simple approach that is compatible with future applications, especially in vivo studies, that may not accommodate washing steps.

### Endocytic Pathways of Cell-Surface Attached DNs

The enhanced cellular uptake of cell-surface attached DNs raised the question of whether cell-surface binding might facilitate the internalization of DNs via nonendocytic processes. To investigate this, we analyzed adhesion-driven particle envelopment using a simple elastic continuum description. Specifically, by modeling lipid membranes as fluid curvature-elastic surfaces, we calculated the energy needed for a membrane to wrap, engulf, and finally internalize a DN, and compared this to the energy gained due to the insertion of cholesterol tags into the membrane (Figure S11). The orientation of DN approaching cell membranes was categorized into horizontal binding and vertical binding depending on the position of overhangs. We found that this adhesion energy was substantially smaller than what would be needed to drive passive uptake (Table S5). Taking nanospheres as an example, the membrane bending energy to engulf a nanosphere is more than about 600 *k*_B_*T*, thus it takes approximately 50 cholesterol tags for the membrane to spontaneously warp the nanosphere (please refer to Experimental Section and Supporting Information for more details on calculations). The number is way larger than what we have on our nanospheres, suggesting that the enhanced internalization of cell-surface attached DNs is achieved through activating stronger endocytosis.

**Figure 6:**
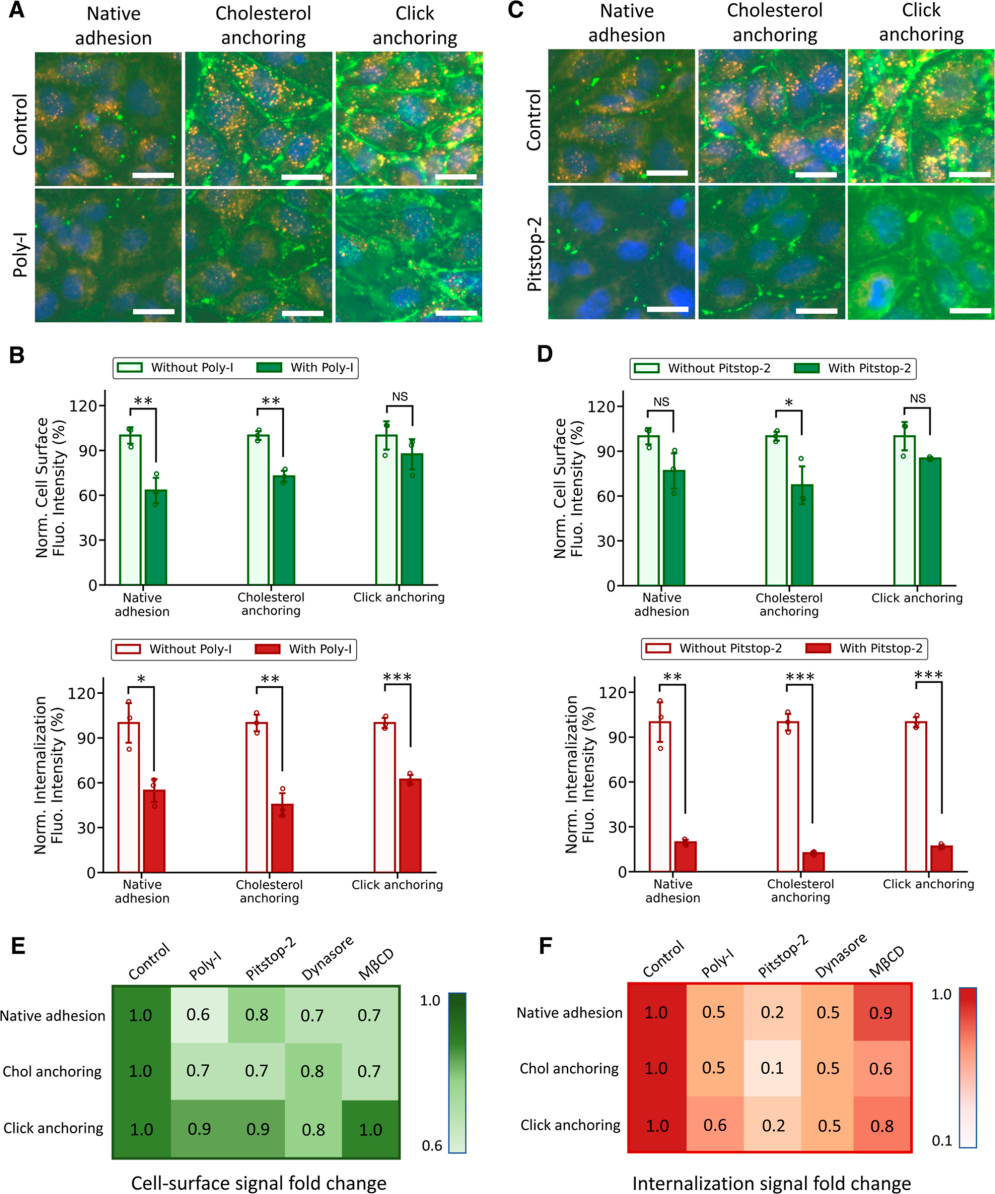
Endocytosis inhibition study of cell-surface attached nanospheres with 4 edge-decorated overhangs (S4). (A) Fluorescence images of cells treated with and without the inhibitor of scavenger receptors, Poly-I. Cells were incubated 30 min at 37 °C with 400 μg/mL of Poly-I, followed by fixation and staining. (B) Quantification of cell-surface and internalization signal intensities for cells incubated with and without Poly-I. (C) Fluorescence images of cells treated with and without the inhibitor of clathrin-mediated endocytosis, Pitstop-2. Cells were incubated 30 min at 37 °C with 10 μM of Pitstop-2, followed by fixation and staining. (D) Quantification of cell-surface and internalization signal intensities for cells incubated with and without Pitstop-2. Heat maps of (E) cell-surface and (F) internalization signal intensities fold change with endocytosis inhibitors. Data were normalized to the control group. In fluorescence images, cell-surface S4 were stained with streptavidin-AF488 (green) and internalized DNs were stained with streptavidin-AF647 (red). Cell nuclei were stained with Hoechst (blue). Data were presented as means ± s.d. in (B) and (D) with *n* = 3. *P ≤ 0.05, **P ≤ 0.01, ***P ≤ 0.001. All scale bars: 25 μm.

Using nanospheres with 4 overhangs (S4), we then sought to understand whether cell-surface attached DNs had similar endocytic pathways as unmodified DNs, or whether there were new pathways involved. Scavenger receptors, clathrin-mediated endocytosis, and caveolin-mediated endocytosis were reported to be responsible for internalizing various shaped DNs.^17,18,29^ Polyinosinic acid (Poly-I) is often used to bind and saturate scavenger receptors. First, cells were introduced with Poly-I at 400 μg/mL and incubated at 37 °C for 30 min. For all three cell-surface binding strategies, including native adhesion, two-step cholesterol anchoring, and click anchoring, the internalization of S4 dropped 40–50% as compared to nontreated cells, which demonstrated the importance of scavenger receptors in internalizing cell-surface-attached S4 (Figure 6A,B). Interestingly, the cell-surface signals also reduced, showing that the treatment of Poly-I affected the ability of S4 to bind to cell membranes. Next, the role of clathrin-mediated endocytosis was investigated by using Pitstop-2 and Dynasore as inhibitors. Pitstop-2 selectively blocks the recruitment of clathrin proteins to the plasma membrane, thereby preventing the formation of clathrin-coated pits and subsequent vesicle formation. We found cells treated with 10 μM Pitstop-2 resulted in only 10–20% of internalized signals as compared to nontreated cells, suggesting the strong relation between clathrin and the internalization of cell-surface-attached S4 (Figure 6C,D). Dynasore blocks the activity of dynamin so that the pinching off of endocytic vesicles from the plasma membrane is inhibited. A 50% decrease in S4 uptake was observed in cells treated with 120 μM dynasore (Figure S12). Further, methyl-β-cyclodextrin (MβCD) is an inhibitor to caveolin-mediated endocytosis by depleting membrane cholesterol, disrupting the structure and function of caveolae. Its relation to S4 internalization appeared to be weaker, with only 10 and 20% decrease in native adhesion and click anchoring, respectively (Figure S13). However, the internalization of S4 in cholesterol anchoring had more than 40% decrease, presumably because of the disruption of cholesterol in membranes. It is worth noting that all four inhibitors negatively impacted the cell-surface binding capability of S4. Moreover, overdose treatment of endocytosis inhibitors may lead to cytotoxicity. For example, we found that 4 mg/mL treatment of Poly-I or 20 μM treatment of Pitstop-2 stressed cells and disrupted the membrane binding of S4. Overall, these results show that the endocytic pathway of membrane-bound DNs is similar to that of unmodified DNs, which is an interdependent process that involves at least scavenger receptors, clathrin- and caveolin-mediated endocytosis (Figure 6E,F).

### Enhanced Cell Nuclear Delivery of Cell-Surface Attached DNs

**Figure 7:**
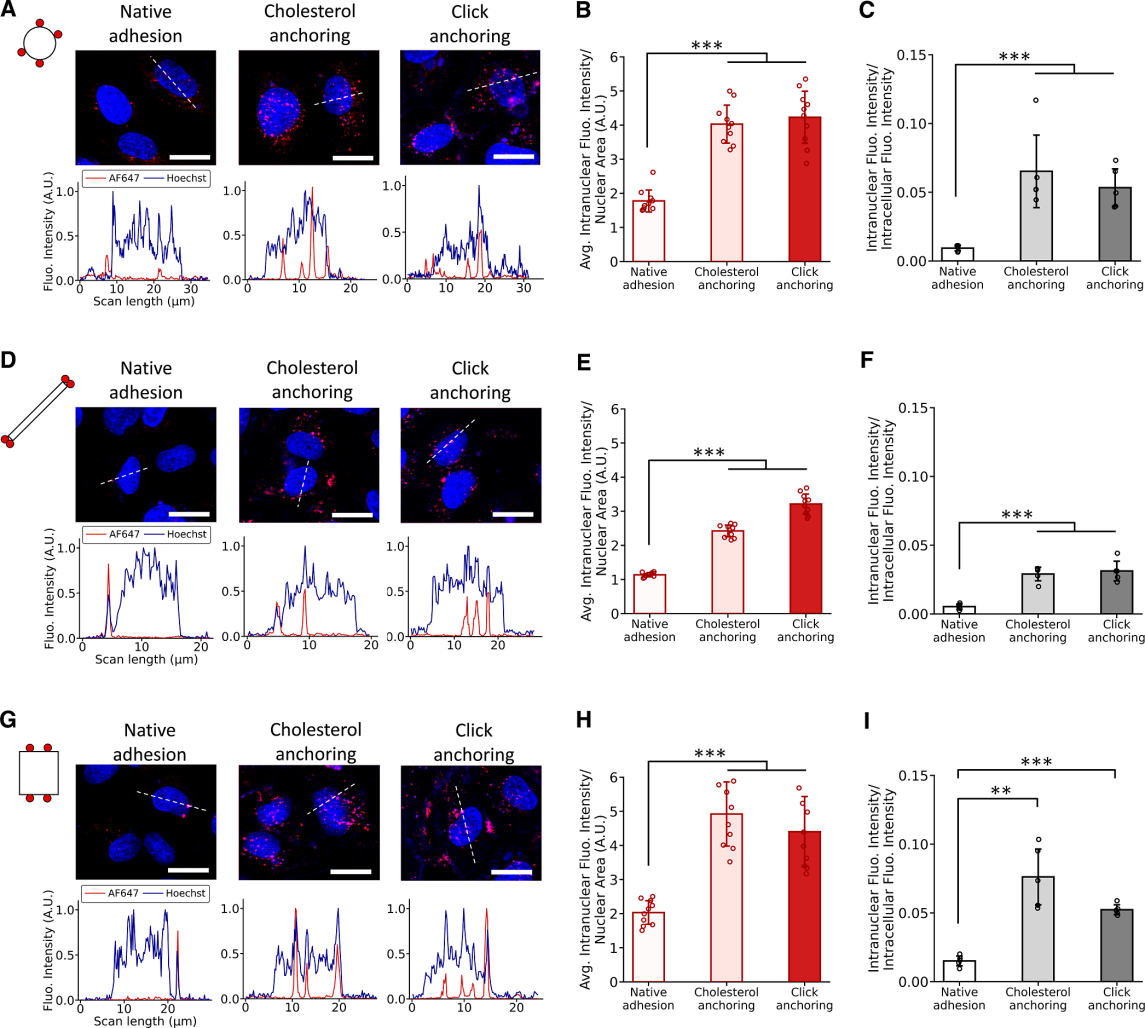
Enhanced cell nuclear delivery of DNs via cholesterol membrane anchoring and click glycocalyx anchoring. Confocal fluorescence images of the intracellular distribution of (A) S4, (D) R4, and (G) T4, and their line-scan profiles. Scale bars: 25 μm. Blue: cell nucleus; red: internalized DNs. Quantification of the average intranuclear fluorescence intensity of (B) S4, (E) R4, and (H) T4 per cell nuclear area. Efficiency of intranuclear delivery of DNs for (C) S4, (F) R4, and (I) T4 by dividing the intranuclear fluorescence intensity by the intracellular fluorescence intensity per cell. Each data point represents the average AF647 intensity per cell nuclear area of all cells in one confocal microscopy image. In all conditions, HUVECs were incubated with DNs for 0.5 h at 37 °C. Data were presented as means ± s.d. **P ≤ 0.01, ***P ≤ 0.001.

Gene-folded DNs have promising applications in gene delivery and therapeutics.^13,14^ To enable these applications, the effective and swift intranuclear delivery of DNs is a central step. Therefore, we conducted an investigation into the capability of cell-surface attached DNs to enter cell nucleus in a short time frame of half an hour. Using confocal fluorescence microscopy images, we identified regions of interest (ROIs) based on the spatial localization of cell nucleus and quantified the fluorescence intensity of AF647-conjugated DNs within ROIs. We then calculated the average fluorescence intensity per cell by dividing the fluorescence intensity within each nucleus by the corresponding nuclei area. In comparison to unmodified DNs, there appeared to be a significantly greater accumulation of DNs within the cell nucleus through both cholesterol membrane anchoring and click glycocalyx anchoring (Figure 7A,D,G). For all shapes of DNs, the increase was at least 2-fold and up to 5-fold (Figure 7B,E,G). We further quantified DN intranuclear delivery efficiency by dividing the intranuclear fluorescence intensity of each cell by its intracellular intensity. Similar trends seemed to hold: cell-surface attachment facilitated the intranuclear delivery efficiency by at least 3-fold and up to 6-fold. It also appears that the shape and size of DNs significantly affect their intranuclear delivery: the nanosphere and nanotile exhibited a more pronounced amount of internalization in cell nucleus compared to the nanorod. The relatively larger size of DNA nanorods presumably limits their ability to enter the nucleus.

While the mechanism by which cell-surface attached DNs enter cell nucleus more efficiently is not clear, a recent study adopted a similar strategy by decorated lipids on DNs for DN delivery.^46^ In the context of the emerging field of gene therapy utilizing gene-folded DNA origami, our method presents a potentially easier and more physiologically relevant route to expedite the delivery of DNs into the cell nucleus. Notably, this approach circumvents the need to compromise cell membranes via electroporation or microinjection.

### Structural Stability and Integrity of DNs

**Figure 8:**
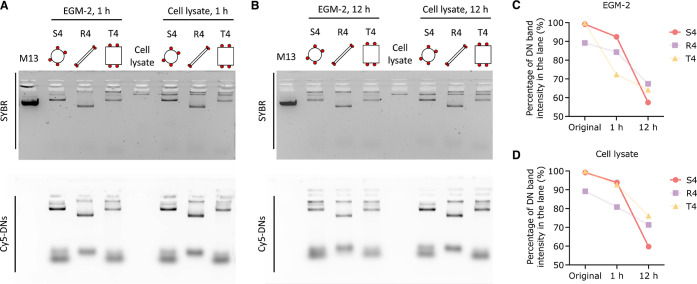
Stability of DNs in cell medium and cell lysate. Agarose gel electrophoresis of S4, R4, and T4 after incubating at 37 °C with EGM-2 and cell lysate for (A) 1 and (B) 12 h. All DNs were conjugated with 14 Cy5 dye molecules. Both SYBR and Cy5 channels were shown. Percentage quantification of DN band intensity in each lane in Cy5 channel with (C) EGM-2 and (D) cell lysate as the incubation buffer.

One challenge in the field of DN cell delivery is the structural stability and integrity of DNs in the cellular environment.^15^ Enzymatic degradation from nucleases, which exist in serum, biological fluids as well as extracellular and intracellular environments, poses a challenge to the integrity of the DNs. Therefore, we examined DN stability in serum-containing cell medium and HUVECs-extracted cell lysate. Three shapes of DNs, each conjugated with 14 Cy5 molecules, were purified and incubated in EGM-2 and cell lysate, respectively, for 1 and 12 h at 37 °C.^47^ Agarose gel electrophoresis characterizations were performed thereafter. Both SYBR and Cy5 channels are shown (Figure 8A,B). Our findings revealed that while a small level of aggregation was observed in the lanes, at 1 and 12 h, all DNs maintained tight and intact bands in gels, demonstrating their resistance against enzymatic degradation from cell medium and nuclease-rich cell lysate in the short term. Further, it seems that the signal depletion of DN bands over time is mainly caused by aggregation rather than degradation or fragmentation. We quantified the percentage of the intact DN band in each lane, and observed a consistent decrease of the signal intensity of DN bands in the Cy5 channel after incubating in EGM-2 and cell lysate, while the percentage of aggregate bands increased (Figure 8C,D). The depletion of DN bands further increased over a longer incubation. We would like to note that the observed DN stability does not ensure their stability inside cells. Many other factors, such as cellular compartmentalization and organelle-specific pH, were not taken into account.^15^ Therefore, further improvements are needed to improve the stability assessment of intracellular DNs.

## Conclusions

The role of nanoparticle shape and size in cellular internalization has been widely acknowledged as an important factor across various materials.^17,27,48^ For example, silver nanoparticles with larger sizes were shown to adhere onto cell membranes and be internalized more effectively.^27^ In another study, Agarwal et al. constructed discoidal and rod-shaped nanoparticles using polyethylene glycol-based hydrogel and found that nanodiscs with large or intermediate sizes exhibited higher levels of cellular internalization.^48^ Interestingly, our study presents a contrasting finding: nanorods were the least preferably internalized among all structures. This observation may be caused by the physical and chemical property differences of the nanoparticles. Further, the change in nanoparticle shape could alter its size and molecular weight for many materials. Since larger structures tend to interact with more membrane receptors, their uptake rates might be higher.^17^ To this point, DNs offer a unique advantage over other materials, as changes in their shape do not always correspond to changes in size and molecular weight when using the same scaffold. This characteristic provides a distinct perspective for evaluating the true impact of shape on cellular internalization. Though the geometry of DNs had an influence on their internalization, we found that the position of ssDNA overhangs may compensate for deficiencies in geometry. Specifically, edge-decorated nanotiles were found to outperform the nanospheres and were the most internalized among all DNs.

Notably, our investigation highlighted a rapid and significant enhancement in the delivery of cell-surface-attached DNs to the cell nucleus by at least 2-fold, presenting promising opportunities for facile nuclear delivery. Interestingly, there seems to exist a positive relation between the cell-surface binding of DNs and their subsequent delivery to the intracellular and intranuclear compartments: DNs that were preferably bound to cell surface, such as nanospheres and nanotiles, have demonstrated better intracellular and intranuclear delivery. Future investigations are needed to elucidate the underlying mechanism responsible for this phenomenon. Although the focus of this study is on enhancing the cellular uptake of DNs, it has been shown that the cross-linking of DNs to form higher-order assemblies may lead to a reduction in DN internalization and an increase in their cell-surface retention time, compared to individual single DN species.^49^ It seems the modulation of DN cellular internalization may be achieved through tuning the interactions between DNs and cell membranes for either speeding up or slowing down the process.

Further, cholesterol membrane anchoring and click glycocalyx anchoring are only two examples of reducing DN-membrane separation for triggering stronger interactions. We expect that cell targeting and cell selectivity can be achieved through targeting DNs to cell-specific membrane proteins. We envision that the cell-surface attaching strategy can be simultaneously utilized when integrating functional molecules such as aptamers and antibodies, on DNs for delivery purposes. Future demonstrations of such applicability are required to showcase the compatibility and impact of other functional molecules.

In summary, we investigated the impact of cell-surface binding on the cellular uptake of DNs, focusing on nanospheres, nanorods, and nanotiles with various ssDNA overhang decorations including overhang number, placement, and spacing. Our findings revealed that both cholesterol membrane anchoring and click glycocalyx anchoring methods significantly enhanced the uptake of DNs by at least 2-fold and up to 8-fold. This improvement was achieved within just half an hour, indicating the rapid speed of uptake. Our study enhances our understanding of the roles of the DN shape and decorations of overhangs in DN cellular uptake, which may provide new insights into the design principles for the development of more effective DN-based delivery systems.

## Experimental Section

### Materials, Reagents, and Equipment

DNA oligos, cholesterol-modified ssDNA, amine-modified ssDNA, and biotin-modified ssDNA (Tables S1–S4) were purchased from Integrated DNA Technologies (Coralville, IA). DBCO-Cy5 and DBCO-Sulfo-NHS-Ester, Sodium Chloride and Paraformaldehyde, and PEG 8000 were purchased from Sigma-Aldrich. Streptavidin-Alexa Fluor 488 and 647 conjugates were purchased from Invitrogen. Roswell Park Memorial Institute (RPMI) 1640 Medium, Dulbecco’s Phosphate-Buffered Saline (PBS) was purchased from Corning. AFM tips were purchased form NanoAndMore (OMCL-AC160TS). ReadyProbes Cell viability imaging kit (Blue/Green) and SYBR Safe DNA gel stain were purchased from Invitrogen. HUVECs, Endothelial Cell Growth Medium-2 BulletKit was purchased from Lonza.

### Synthesis and Purification of Biotinylated DNs

All DNs were folded from M13mp18 scaffold (Bayou Biolabs) together with staple strands (Tables S1–S4) through custom annealing ramps. The caDNAno or vHelix design of DNs and the placement of overhangs are presented in Figure S1. For annealing, 20 nM of the scaffold and 100 nM of the staples were mixed in TAE buffer with 12.5 mM MgCl_2_ (nanorods and nanotiles) and with 20 mM MgCl_2_ for nanospheres. The biotin-labeled strands were added preannealing with the same concentration as other staples. Cholesterol-conjugated DNs used 3× excess of cholesterol-conjugated staples. The mixture for synthesizing nanotiles was heated to 90 °C for 5 min and gradually cooled down to 4 °C within 4 h. Specifically, 90–70 °C, 0.1 °C per 6 s; 70–45 °C, 0.1 °C per 30 s; 45–30 °C, 0.1 °C per 6 s; 30–4 °C, 0.1 °C per 3 s. The mixture for synthesizing nanospheres and nanorods was heated to 80 °C for 5 min and gradually cooled down to 4 °C within 45 h. Specifically, 80–65 °C, 0.1 °C per 24 s; 65–24 °C, 0.1 °C per 378 s; 24–4 °C, 0.1 °C per 18 s. After annealing, the contents were collected and precipitated by centrifugation at 10 500*g* for 25 min in a buffer containing 7.5% PEG 8000, 10 mM MgCl_2_, 255 mM NaCl, 22.5 mM Tris, 10 mM acetic acid, and 1 mM EDTA. The supernatant was removed and the remaining was resuspended to 5 nM using PBS buffer.

### Characterizations of DNs

For agarose gel electrophoresis, around 10 μL of 10 nM purified DN samples were first incubated in PBS buffer for 30 min at 37 °C, and then analyzed by electrophoresis in 2% agarose gel in TBE buffer with 12.5 mM MgCl_2_ at 90 V and room temperature (RT) for 1 h. The gels were stained with 1× SYBR Safe DNA gel stain and imaged with Biorad ChemiDoc Imaging System. For AFM characterization, 10 μL of purified DN samples (1–2 nM in concentration) in TAE with 12.5 mM MgCl_2_ was deposited onto a freshly cleaved mica surface, incubated at RT in a humid chamber for 5 min. The sample was washed three times with 20 μL DI water and thoroughly blow-dried with nitrogen after each washing. AFM scans were performed using NX10 AFM system with OMCL-AC160TS tips preloaded onto a wafer (Park Systems Corp.), in noncontact mode (NCM).

### Synthesis of DBCO-ssDNA

The NH_2_-ssDNA oligos were incubated overnight in PBS with DBCO-sulfo-*n*-hydroxysuccinimidyl ester (DBCO-Sulfo-NHS-Ester) at a 1:10 molar ratio under agitation at RT. Thirty min After incubation, the reaction mixture was dialyzed five times against PBS using Amicon Ultra Centrifugal filters (molecular weight cutoff, 3 kDa) to remove unconjugated DBCO-Sulfo-NHS-Ester. The above-described conjugation and purification procedures were repeated twice in total to increase the conjugation efficiency. The final concentration of DBCO-ssDNA was adjusted to 500 μM with PBS.

### Cell Culture

HUVECs were cultured in Endothelial Cell Growth Medium-2 (EGM-2) at 37 °C with 5% CO_2_. HUVECs of passage 3–5 were plated into 96-well culture plates at a density of 50 000 cells per well, 1 day prior to the experiment.

### Two-step Cholesterol Membrane Anchoring

First, cholesterol-ssDNA was diluted to 0.5 μM in PBS and administered to cells followed by 1 h incubation at 37 °C.^30^ After incubation, cells were washed four times with PBS. Next, 5 nM of DNs bearing complementary ssDNA overhangs were introduced to cells followed by 30 min incubation. Cells were then washed and fixed, and the cell-surface DNs and internalized DNs were visualized via dual-color streptavidin staining.

### Two-step Click Glycocalyx Anchoring

First, azide ligands were labeled on cell-surface glycocalyx through metabolic labeling.^26^ Specifically, azido monosaccharide, *N*-azidoacetylmannosamine-tetraacylated (Ac4ManNAz, stock in DMSO) was diluted in culture medium to a final concentration of 50 μM and administered to cells for 2 days prior to the experiment. Next, cells were incubated with DBCO-ssDNA at 50 μM in PBS for 1 h at 37 °C to allow for DBCO-azide click conjugation. After incubation, cells were washed four times with PBS. 5 nM of DNs bearing complementary ssDNA overhangs were then introduced to cells followed by 30 min incubation. Next, cells were washed and fixed, and the cell-surface DNs and internalized DNs were visualized via dual-color streptavidin staining.

### One-step Cholesterol Membrane Anchoring

5 nM of DNs with direct conjugation of cholesterol tags were introduced to cells followed by 30 min incubation at 37 °C.^44,45^ Cells were then washed and fixed, and the cell-surface DNs and internalized DNs were visualized via dual-color streptavidin staining.

### Dual-Color Streptavidin Staining of Cell–Surface Bound and Internalized DNs

After incubating with DNs, cells were washed four times using PBS. Next, cells were fixed with 4% PFA, washed, and first stained with streptavidin-Alexa Fluor 488 in 1% BSA in PBS for 30 min to visualize cell-surface DNs. Cells were then washed four times with PBS. 0.5% Triton-X was used to permeabilize cell membranes by incubating cells for 15 min at RT. The internalized DNs were then stained by incubating the permeabilized cells with the streptavidin-Alexa Fluor 647 in 1% BSA in PBS for 30 min at RT. Cells were washed again before imaging.

### Quantitative Image Analysis of Internalized DNs

To minimize background noise caused by nonspecific cell internalization of fluorophores, we performed image analysis to account for internalized DN signals more accurately. Specifically, internalized signals were identified by constructing structuring elements and performing morphological transformations (Figure S4). The intensities of postprocessed images were then calculated by summing up the intensity of all pixels. For cell-surface signals, the intensity of each image was directly computed. The intensity of autofluorescence from cells and the buffer was subtracted.

### Flow Cytometry Measurement for Quantifying Cell–Surface and Internalization Fluorescence Intensity

HUVECs were cultured in 6 wells plates until reaching at least 70% confluency. For each condition, three wells of cells were prepared. Initiator immobilization and DNs binding were conducted in well plates. Next, 0.5 mL of 0.25% trypsin was added to each well to detach cells by incubating cells at 37 °C for 2 min. 0.5 mL RPMI + 10% containing FBS buffer was used as neutralizing medium. Cells were then collected and washed for fixation and dual color staining. The concentration of cells was adjusted to 1 million per ml for flow cytometry measurement.

### Adhesion-Driven Envelopment of DNs

To study if the internalization of membrane-bound DNs could be a spontaneous membrane wrapping process driven by adhesion, we calculated the adhesion energy provided by the insertion of cholesterol tags into lipid membranes, and the energy needed for a membrane to wrap, engulf, and finally internalize a DN. Here, lipid membranes were modeled as a simple elastic continuum. The adhesion energy provided by cholesterol insertion is *E*_adhesion_ = *n*(*x* × *k*_B_*T*), where n is the number of cholesterol tags inserted into the membrane and *x* × *k*_B_*T* is the adhesion gained from the insertion of one cholesterol. A membrane’s bending energy can be described by the classical curvature-elastic model due to Helfrich.^50^ For nanorods and nanotiles, we considered two possible orientations when they interact with cell membranes, vertically and horizontally (Figure S11). We found that for all DNs, the adhesion energy was substantially smaller than what would be needed to drive passive uptake (Table S5). Let us look at the case of nanospheres to illustrate the point. Suggested by previous theoretical and experimental calculations, we took the bending modulus of the cellular membrane κ to be 25*k*_B_*T* and *x* = 13.^7^ Then the membrane bending energy is , independently of sphere radius. For spontaneous wrapping to happen, *E*_bending_ must be smaller than or equal to *E*_adhesion_. This shows that *n* ≥ 48, or in other words, that we would need many more cholesterol anchors to drive successful wrapping and internalization than we actually use. This suggests that active cellular processes assist particle uptake, such as for instance endocytosis.

### Inhibition of Endocytic Pathways Study

Inhibitors were diluted using EBM-2 to a certain concentration: Poly-I at 400 μg/mL, Pitstop-2 at 10 μM, Dynasore at 120 μM and MβCD at 300 nM. Cells were first incubated with each inhibitor for 30 min at 37 °C. Then cells were washed and administrated with cholesterol anchors for two-step cholesterol anchoring, or DBCO-ssDNA for two-step click anchoring. After incubation, cells were washed and incubated with DNs for 30 min at 37 °C, followed by washing, fixation, and staining. Fluorescence microscopy images of cells were taken and assessed for quantification.

### Quantification of the Intranuclear Delivery of Cell–Surface Bound DNs

Three shapes of DNs with 4 overhangs (S4/R4/T4) were labeled with AF-647 and were introduced to HUVECs in a 96 well plate, respectively. Native adhesion, cholesterol membrane anchoring, and glycocalyx anchoring were studied. Cells were incubated with DNs at 37 °C for 30 min. Cell-surface DNs were removed using DNase I at 20 U. Fluorescence images were taken using confocal fluorescence microscopy. Next, images were analyzed using ImageJ and MATLAB to calculate the signal intensity in the cell nuclei area. Specifically, cell nucleus ROIs were selected, which were slightly smaller than cell nuclei with the purpose of minimizing artifacts or the presence of DNs on the surface of the nucleus. Then the fluorescence intensity value was divided by the corresponding nuclear area. For quantifying the intranuclear delivery efficiency, fluorescence intensity in the cell nuclear area per cell was divided by the fluorescence intensity in the whole cell area.

### DNs Stability Assay

Cell lysate was prepared using a previously described lysis buffer containing 50 mM Tris-HCI, 150 mM NaCl, 0.1% SDS, 0.5% deoxycholic acid and 1x protease inhibitor.^47^ HUVECs were initially seeded in 6-well plates. After reaching confluency, cells were trypsinized and resuspended with cell lysis buffer (500 μL for every one million cells). The resulting contents were collected in microcentrifuge tubes, agitated on a shaker with ice for 20 min, and then centrifuged at 16 100*g* for 30 min at 4 °C. The supernatants were collected thereafter. Three edge-decorated DNs were first purified at 20 nM in PBS and then diluted with cell lysate to around 5 nM. After incubating for 1 and 12 h at 37 °C, respectively, DNs were characterized by agarose gel electrophoresis. To avoid errors caused by concentration and volume bias between groups, we quantified the DN bands by calculating the ratio of the intensity of DN bands to the total intensities of all bands within each lane.

### Statistics and Reproducibility

Quantitative data were displayed as means ± s.d. Statistical significances were determined using one-way analysis of variance (ANOVA) with posthoc Tukey’s Test. All cell experiments were repeated independently at least three times.

## Supporting Information Available

The design and sequences of DNs, real-value quantification of the cell-surface binding and cellular uptake of DNs, the cytotoxicity of DNs, endocytosis inhibition study using dynasore and MβCD, and the calculation of membrane deformation energy for spontaneous membrane wrapping of DNs PDF
