# Supplementary material for: Cell–Surface Binding of DNA Nanostructures for Enhanced Intracellular and Intranuclear Delivery
Source: ACS Appl Mater Interfaces. 2024 Mar 18;16(13):15783–97. doi: 10.1021/acsami.3c18068 (PMC10995898; doi:10.1021/acsami.3c18068)
Supplement: Supplementary file 1 — am3c18068_si_001.pdf [file am3c18068_si_001.pdf]

Supporting Information

Cell-surface Binding of DNA Nanostructures for Enhanced Intracellular and Intranuclear Delivery

Weitao Wang[#,†], Bhavya Chopra[#,‡], Vismaya Walawalkar[†], Zijuan Liang[†]

Rebekah Adams[†], Markus Deserno[¶], Xi Ren[‡,†] and Rebecca E. Taylor[,†,‡,§]*

[†] Department of Mechanical Engineering, Carnegie Mellon University, Pittsburgh, Pennsylvania, 15213, United States

[‡] Department of Biomedical Engineering, Carnegie Mellon University, Pittsburgh, Pennsylvania, 15213, United States

[¶] Department of Physics, Carnegie Mellon University, Pittsburgh, Pennsylvania, 15213, United States

[§] Department of Electrical and Computer Engineering, Carnegie Mellon University, Pittsburgh, Pennsylvania, 15213, United States

# W.W. and B.C. contributed equally to this paper.

∗ Corresponding author

E-mail: bex@andrew.cmu.edu

List of Supplementary Information:

Figure S1. The caDNAno designs of DNs.

Figure S2. Yield of DNs by nanodrop measurement.

Figure S3. Administration of strep-AF488 to pre-permeabilized cells. Supplementary

Figure S4. Fluorescence image processing by using morphological transformation and adaptive thresholding to reduce background noise.

Figure S5. The effect of cell membrane modification with cholesterol on DN cellular internalization.

Figure S6. Absolute value quantification of cell-surface, internalization signals and internalization efficiency using cholesterol anchoring.

Figure S7. Cytotoxicity of DNs on treated cells.

Figure S8. Absolute value quantification of cell-surface, internalization signals and internalization efficiency using click anchoring.

Figure S9. DN concentration-dependent membrane binding and uptake of DNs.

Figure S10. Cholesterol anchor concentration-dependent membrane binding and uptake of DNs.

Figure S11. Schematic illustrations of membrane wrapping of DNs.

Figure S12. Endocytosis inhibition study by using dynasore to inhibit clathrin-mediated endocytosis.

Figure S13. Endocytosis inhibition study by using MβCD to inhibit caveolin-mediated endocytosis.

Table S1. List of functional DNA oligos.

Table S2. List of DNA oligos for DNA nanospheres.

Table S3. List of DNA oligos for DNA nanorods.

Table S4. List of DNA oligos for DNA nanotiles.

Table S5. Calculation of membrane deformation energy needed for the spontaneous membrane wrapping of DNs.

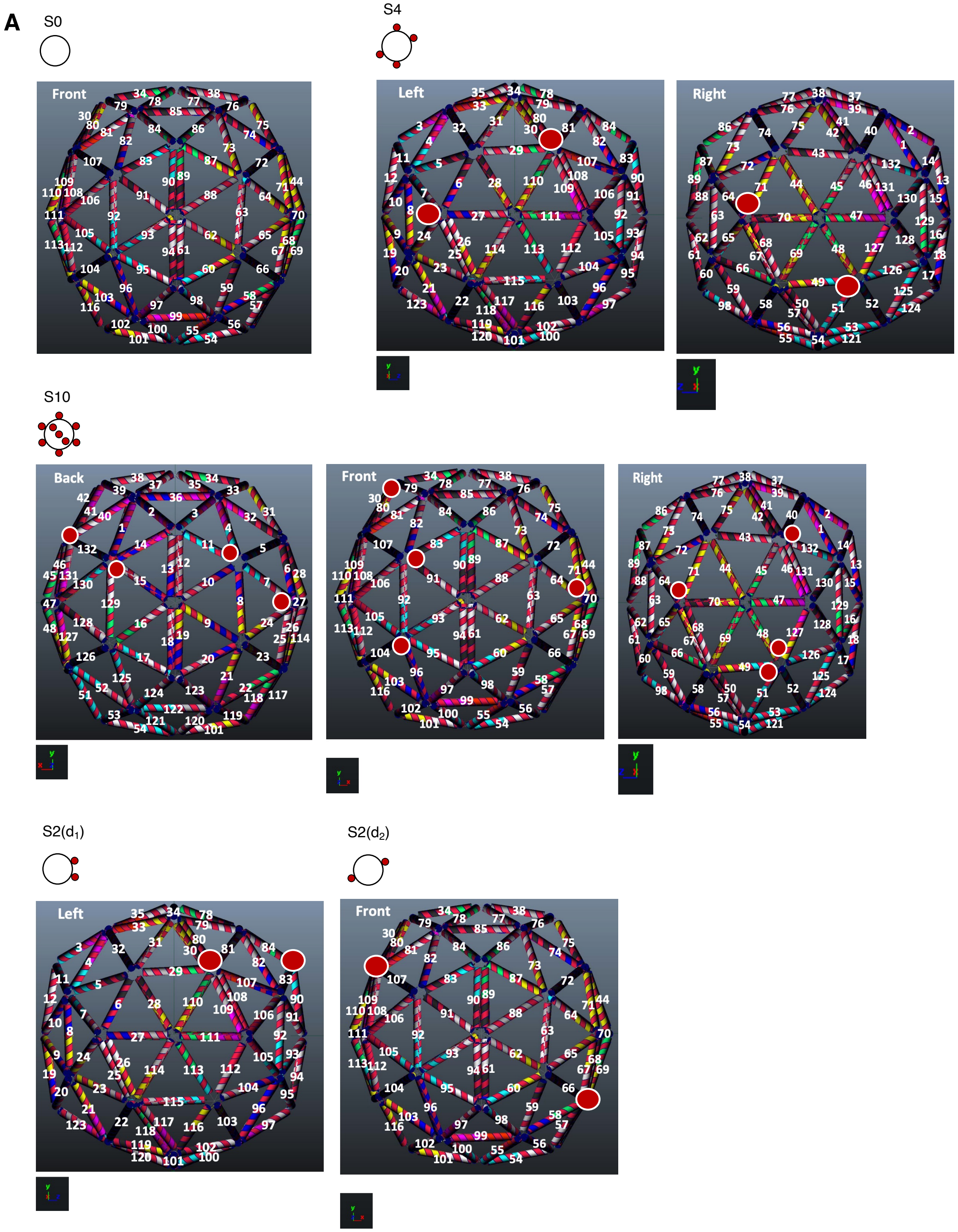
A
S0
Front
S4
Left
Right
S10
Back
Front
Right
S2(d1)
Left
S2(d2)
Front

B

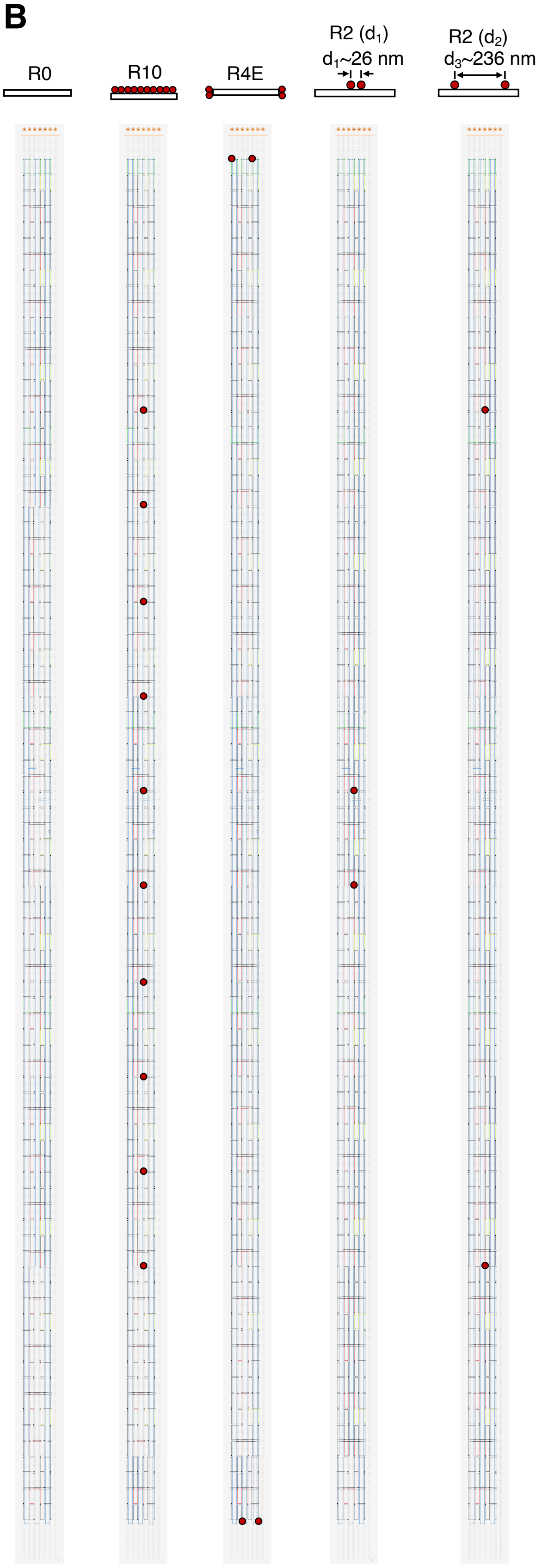

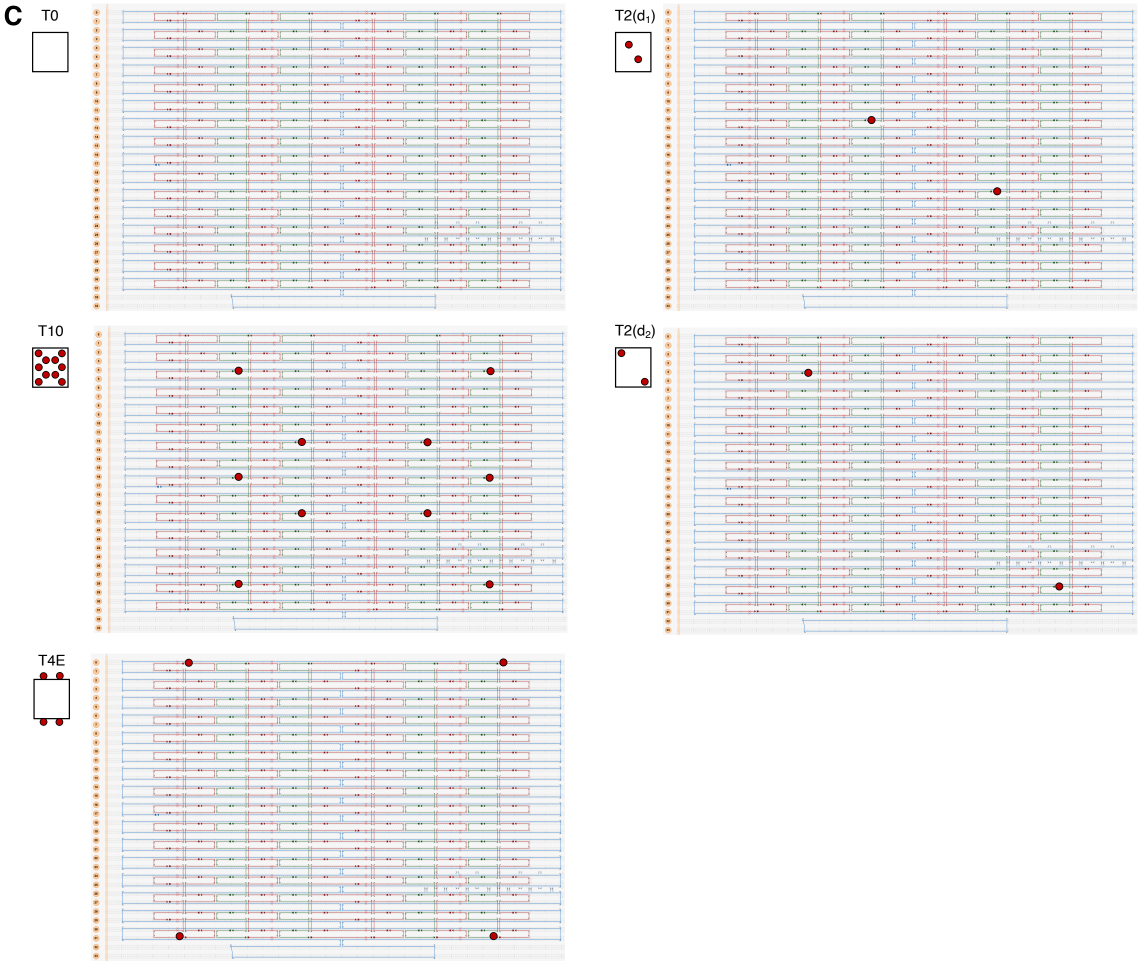

Figure S1. The structural design of DNs: (A) vHelix design of the DNA nanospheres, and caDNAno design of (B) nanorods and (C) nanotiles. Red dots represent the overhang positions.

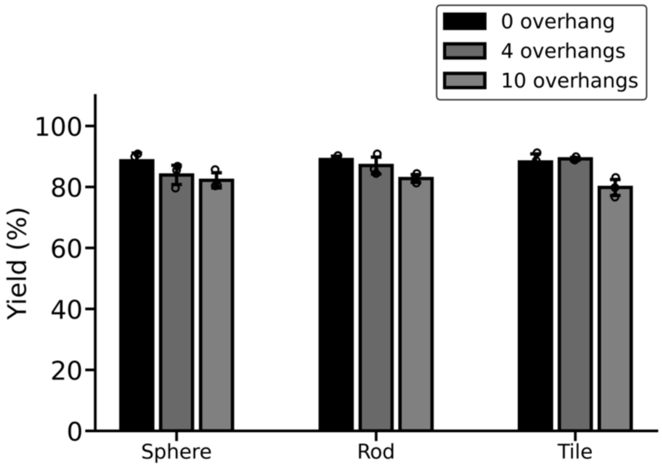

Figure S2. Yield of DNs by nanodrop measurement.

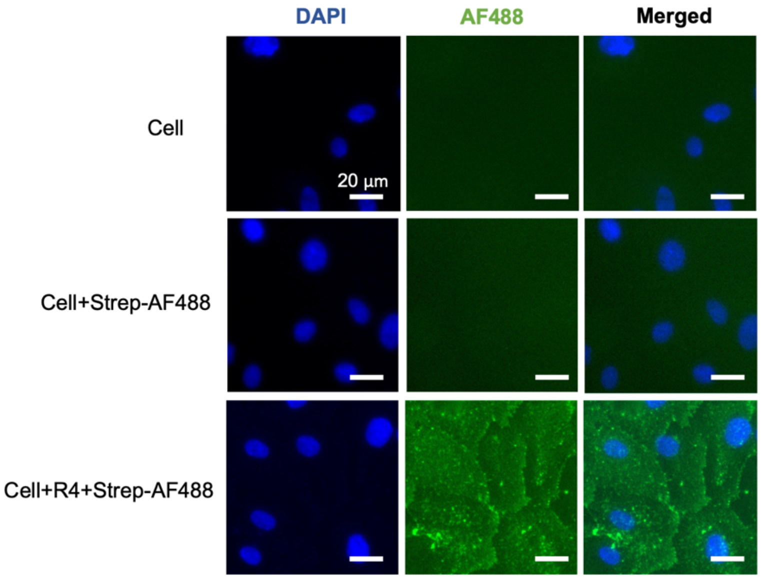

Figure S3. Administration of strep-AF488 to pre-permeabilized cells. Blue: cell nucleus. Green: streptavidin-AF488.

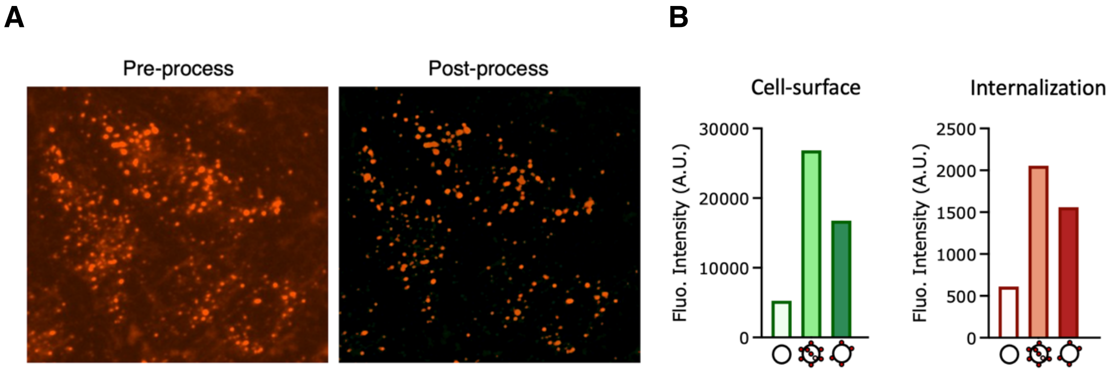

Figure S4. Fluorescence image processing by using morphological transformation and adaptive thresholding to reduce background noise.[1,2] (A) A comparison between pre-processed and post-processed internalization signals (red). (B) Flow cytometry quantification of the cell-surface attachment and internalization of S0, S10 and S4 using cholesterol anchoring. The average values of fluorescence intensities were taken.

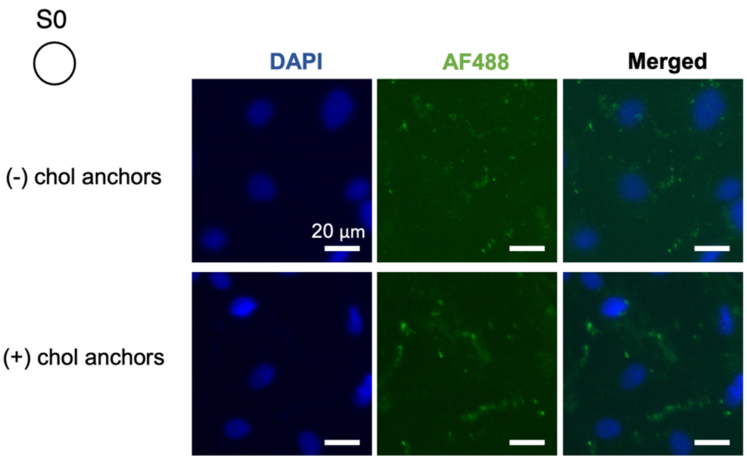

Figure S5. The effect of cell membrane modification with cholesterol on DN cellular internalization. Blue: cell nucleus. Green: streptavidin-AF488.

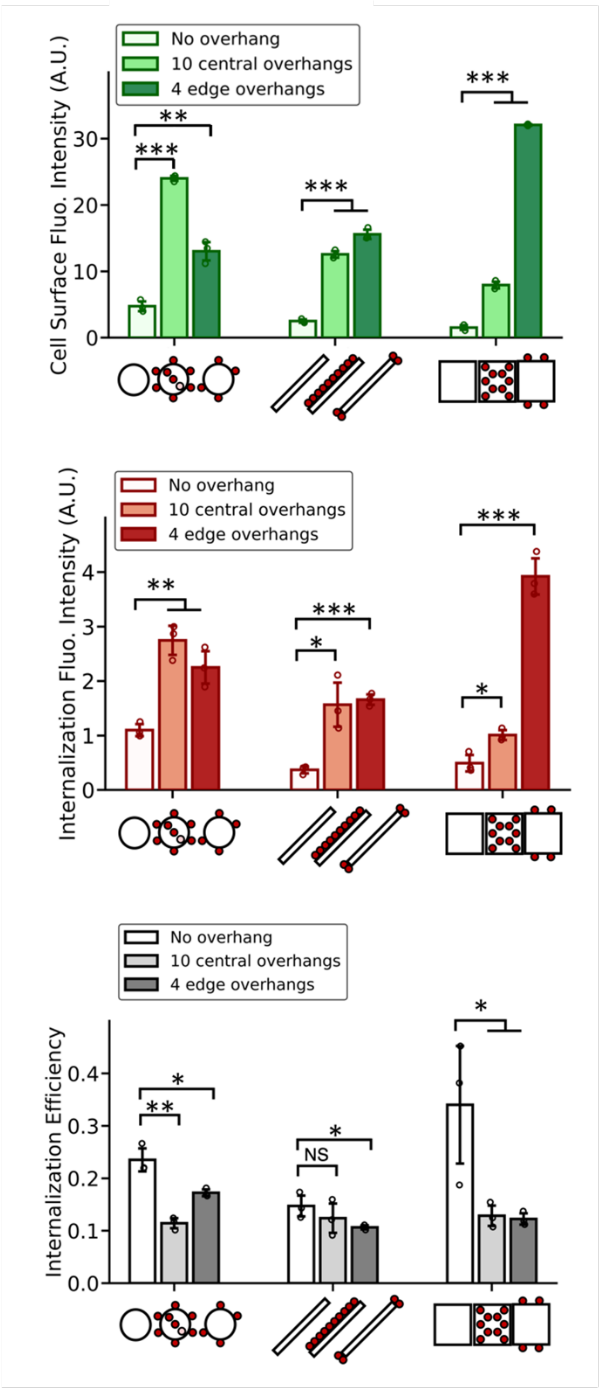

Figure S6. Quantification of cell-surface, internalization signals and internalization efficiency using cholesterol anchoring by absolute values. Data were presented as means ± s.d. with n=3. *P ≤ 0.05, **P ≤ 0.01, ***P ≤ 0.001.

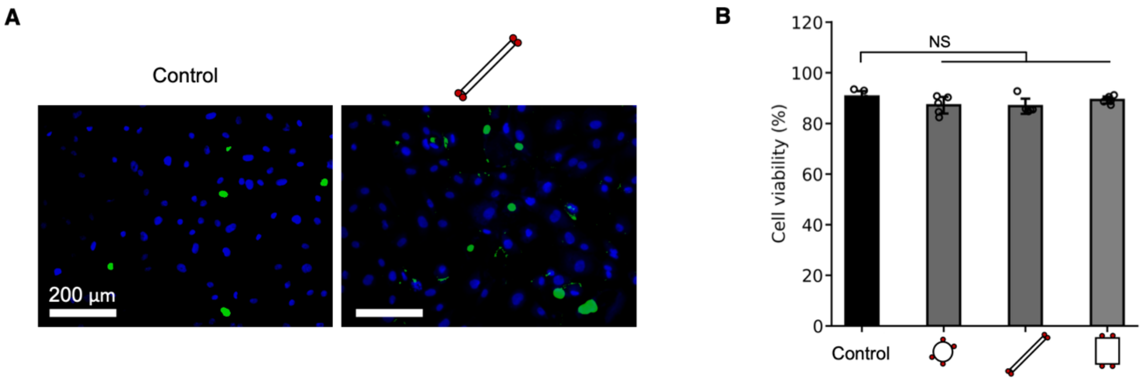

Figure S7. Cytotoxicity of DNs on treated cells. (A) Representative fluorescence images of cell viability examination by comparing pre-treated cells and T4E treated cells. Blue: all cells. Green: dead cells. (B) Quantification of cell viability after cells were incubated with three 4-edge-decorated DNs.

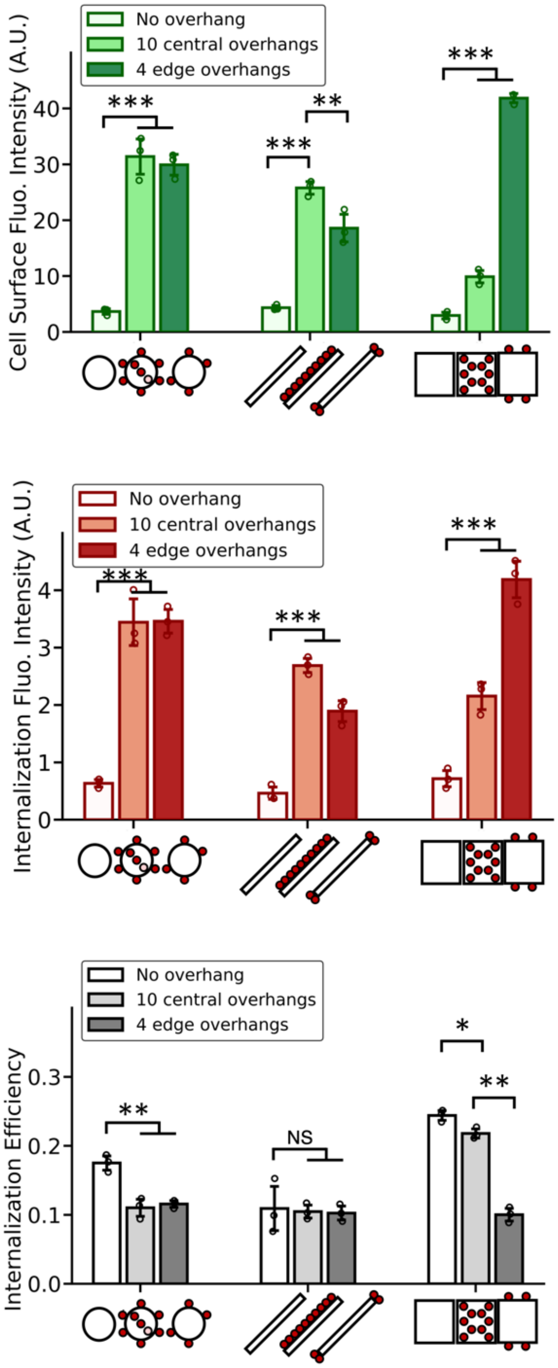

Figure S8. Quantification of cell-surface, internalization signals and internalization efficiency using click anchoring by absolute values. Data were presented as means ± s.d. with n=3. *P ≤ 0.05, **P ≤ 0.01, ***P ≤ 0.001.

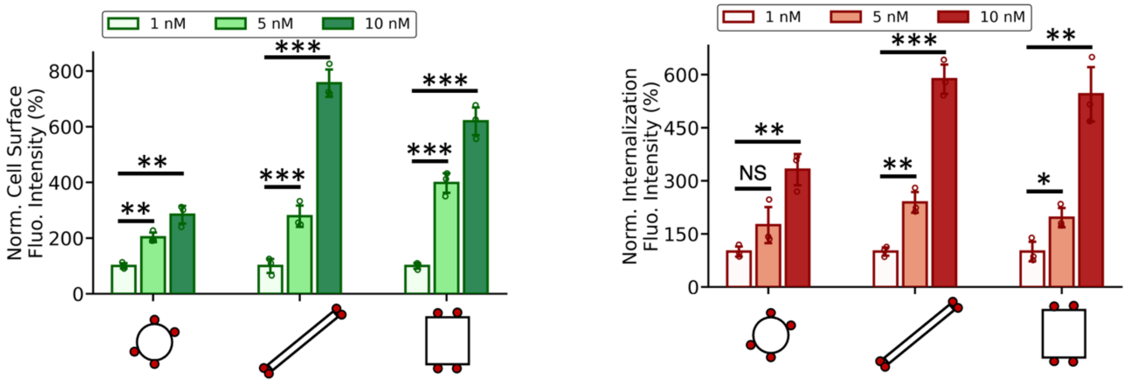

Figure S9. DN concentration-dependent membrane binding and uptake of DNs. The concentration of DNs were changed from 1 nM, 5 nM to 10 nM. All data were normalized to 1 nM group. *P ≤ 0.05, **P ≤ 0.01, ***P ≤ 0.001.

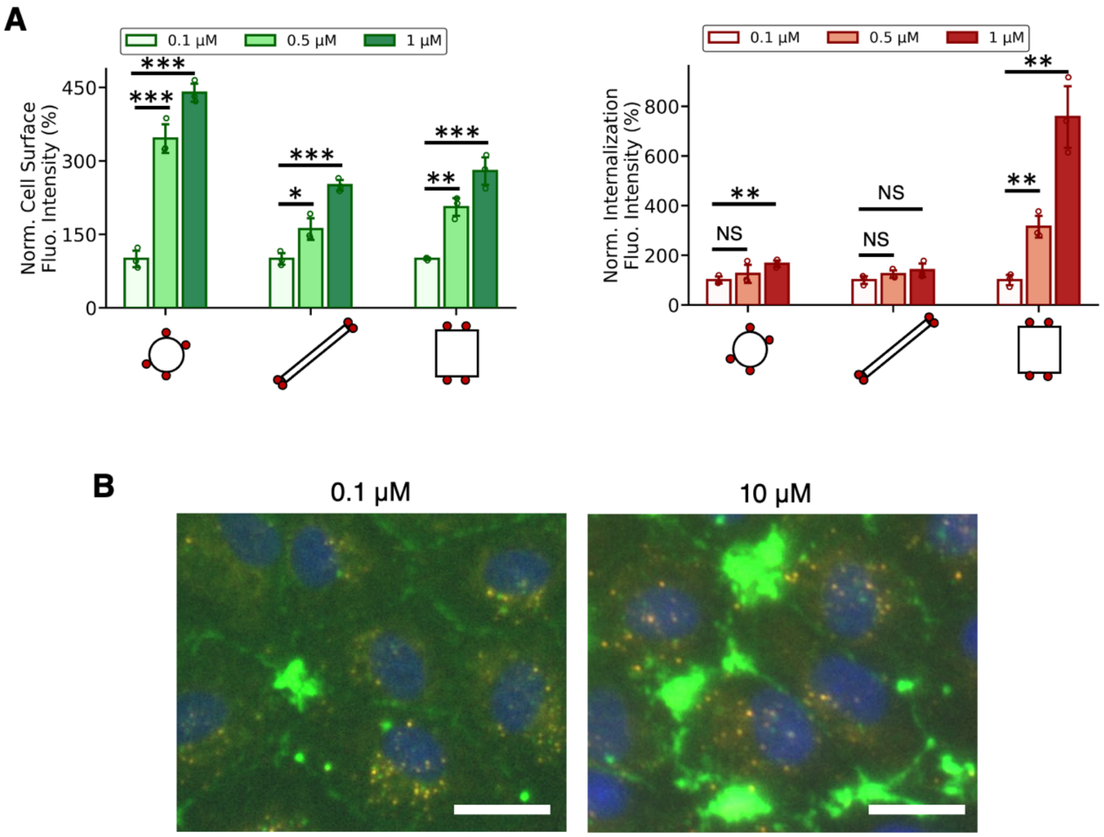

Figure S10. (A) Cholesterol anchor concentration-dependent membrane binding and uptake of DNs. The concentration of cholesterol anchors were changed from 0.1 µM, 0.5 µM to 1 µM. All data were normalized to 0.1 nM group. (B) Aggregations triggered by a high concentration of cholesterol anchors. Blue: cell nucleus. Green: streptavidin-AF488. Scale bars: 10 µm. *P ≤ 0.05, **P ≤ 0.01, ***P ≤ 0.001.

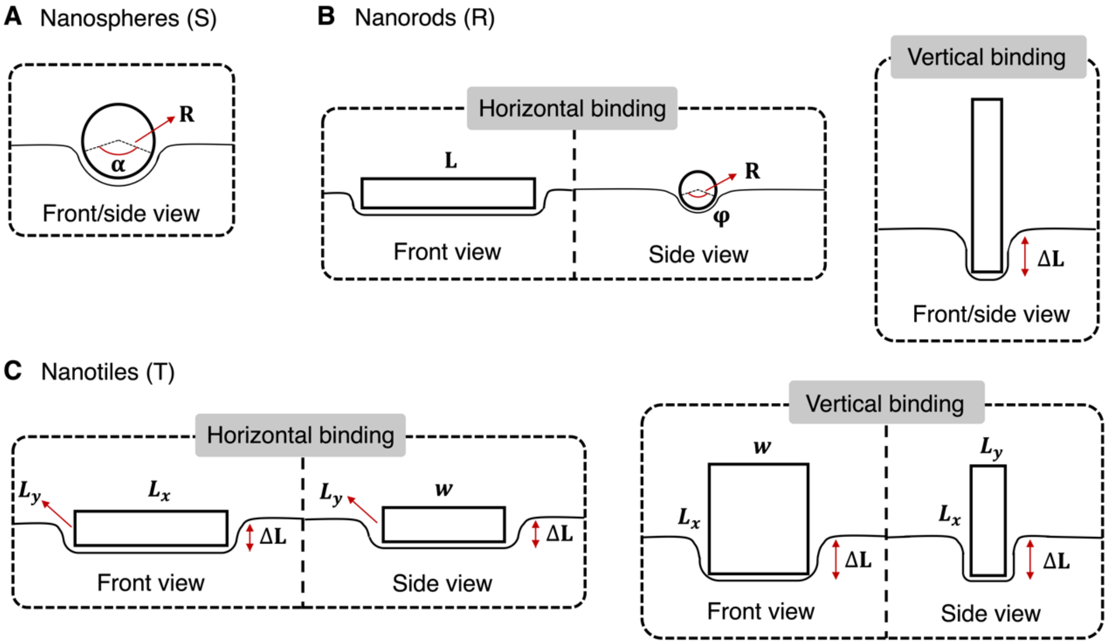

Figure S11. Schematic illustrations of membrane wrapping of DNs, including (A) DNA nanospheres, (B) nanorods and (C) nanotiles. For each DN, two approaches of membrane wrapping were analyzed, including DN horizontal binding and vertical binding to membranes. φ and ΔL denote the angle and length of the DN that had been wrapped by the lipid membranes, respectively. The diameter of nanospheres is R. The diameter of the nanorod is R and its length is L. The dimension of the nanotile is $L_x * L_y * w$.

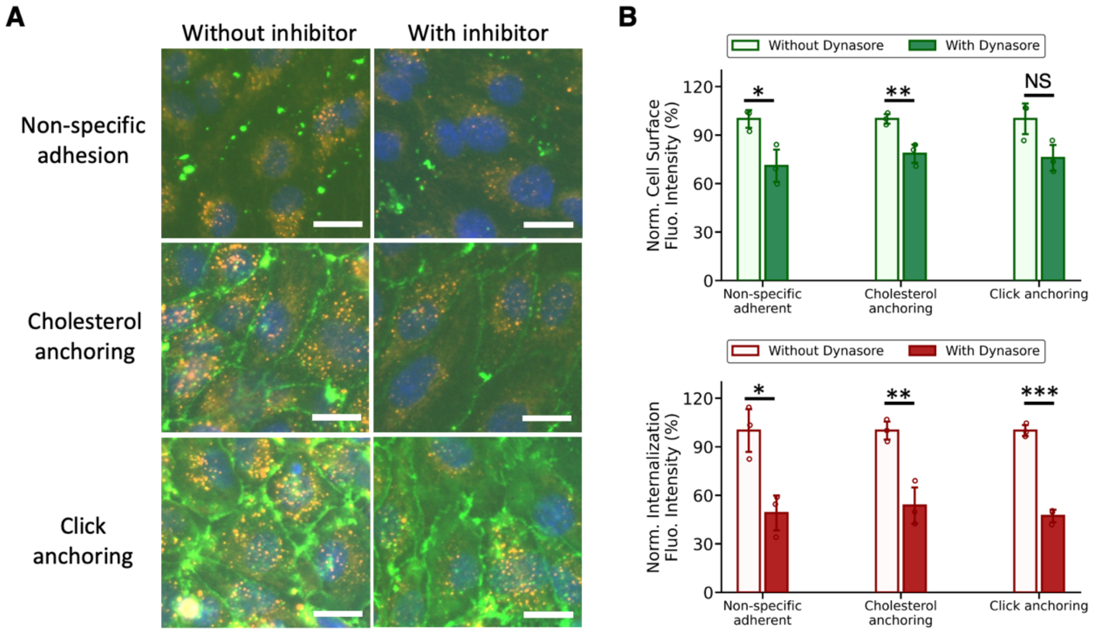

Figure S12. Endocytosis inhibition study by using Dynasore to inhibit clathrin-mediated endocytosis. (A) Fluorescence images after incubating cells with and without Dynasore. Cells were incubated 30 min at 37°C with 120 µM of Dynasore, followed by fixation and staining. Blue: cell nucleus. Green: streptavidin-AF488 (cell surface DNs). Red: streptavidin-AF647 (internalized DNs). All scale bars: 10 µm. (B) Quantification of cell-surface and internalization signal intensities for cells incubated with and without Dynasore. $^{*}P \leq 0.05$, $^{**}P \leq 0.01$, $^{***}P \leq 0.001$.

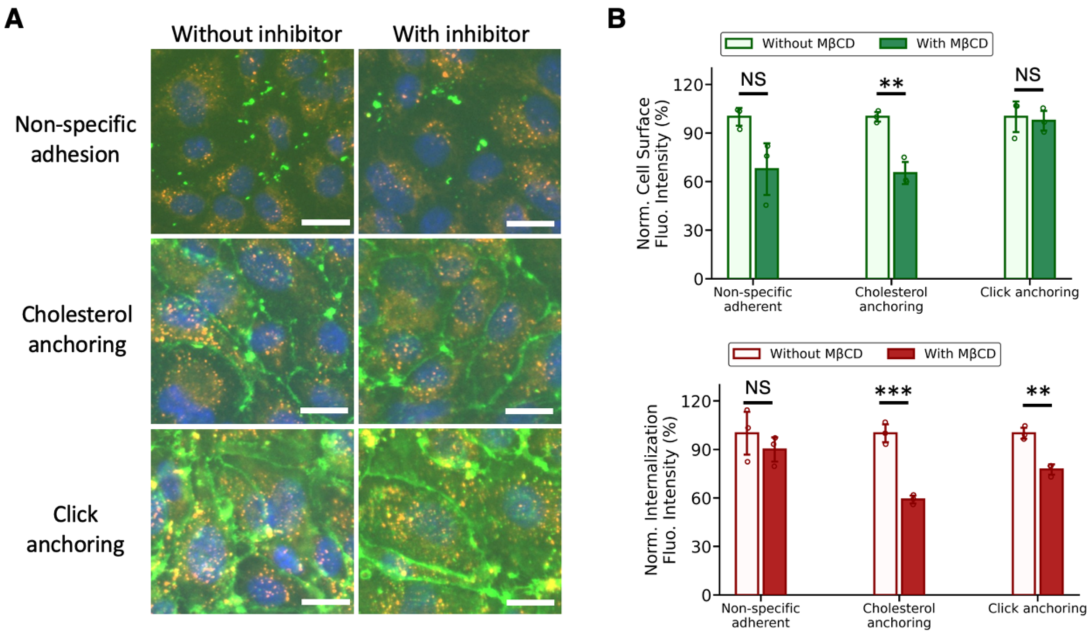

Figure S13. Endocytosis inhibition study by using MβCD to inhibit caveolin-mediated endocytosis. (A) Fluorescence images after incubating cells with and without MβCD. Cells were incubated 30 min at 37°C with 300 nM of MβCD, followed by fixation and staining. Blue: cell nucleus. Green: streptavidin-AF488 (cell surface DNs). Red: streptavidin-AF647 (internalized DNs). All scale bars: 10 µm. (B) Quantification of cell-surface and internalization signal intensities for cells incubated with and without MβCD. $^{**}P \leq 0.01$, $^{***}P \leq 0.001$.

Table S1. List of functional DNA oligos.

Name of oligos	Sequence
ssDNA on DNs for two-step membrane binding (both cholesterol membrane anchoring and click glycocalyx anchoring)	5'/TT CAGTCAGTCAGTCAGTCAGT/3'
ssDNA on DNs for attaching biotin	5'/TT GAGAGCAGACCTGGAACTCG/3'
Complementary ssDNA conjugated with biotin	5'/BiosG/TT CGAGTTCCAGGTCTGCTCTC/3'
Cholesterol-ssDNA anchors for immobilizing cell membranes in two-step cholesterol anchoring	5'/Chol-TEG/ACTGACTGACTGACTGACTG/3'
5'NH_2-ssDNA for synthesizing DBCO-ssDNA anchors in click anchoring	5'/AmMC6/TT ACTGACTGACTGACTGACTG/3'
Cholesterol sequence for one-step cholesterol membrane binding	5'/Chol-TEG/AAACTGACTGACTGACTG/3'
Shielding adjacent ssDNA on DNs for one-step cholesterol membrane binding	5'/TT CAGTCAGTCAGTCAGTTTCCATCA/3'

Table S2. List of DNA oligos for DNA nanospheres

CCACCCTCAGAGCCACCACCCTCATAGCTATCTTACCGAAGCCCT			
AGCCACCACCGGAACCGCCTCCCACTATATGTAAATGCTGATGCAAATCC			
TTATTAGCGTTTGCCATCTTTTCATGTTAGCAAACGTAGAAAAT			
CGTCAGACTGTAGCGCGTTTTCAATCATATGCGTTATACAAATTCTTACC			
GATAGCAGCACCGTAATCAGTAGAAAGGTGAATTATCACCGTCACCG			
ACCATTACCATTAGCAAGGCCATGTTCAGCTAATGCAGAACGCGC			
ACTTGAGCCATTTGGGAATTAGATTTCATCGTAGGAATCATTAC		yes (center)	yes (edge)
AATATTGACGGAAATTATTCATTTATAAAAGAAACGCAAAGACACCA			
AGCGCCAAAGACAAAAGGGCGACATTCGAGCGTCTTTCCAGAGCCTAATT			
CGGAATAAGTTTATTTTGTCACAGGAATACCCAAAAGAACTGGCATG			
ACATACATAAAGGTGGCAACACGACAGAATCAAGTTTGCCTTTAG		yes (center)	
ATTAAGACTCCTTATTACGCAGTAGCCGAACAAAGTTACCAGAAGGA			
AACCGAGGAAACGCAATAATAACAATTGAGTTAAGCCCAATAATAAG			
TTTTAAGAAAAGTAAGCAGATATAATCAAAATCACCGGAACCAG			
AGCAAGAAACAATGAAATAGCAATACCGTTCCAGTAAGCGTCATACATGG		yes (center)	
ATATCAGAGAGATAACCCACAAGCAAAAATGAAAATAGCAGCCTTTA			
ATTAACTGAACACCCTGAACAGATTAGCGGGGTTTTGCTCAGTAC			
CAGAGAGAATAACATAAAAACAGATATTATTTATCCCAATCCAAATA			
AGAAACGATTTTTTGTTTAACGTATCAATAGAAAATTCATATGGTTTACC			
TGCCAGTTACAAAATAAACAGCCAGGTTTAGTACCGCCACCCTC			
ATCCTGAATCTTACCAACGCTAACAGATATAGAAGGCTTATCCGGTA			
TGAAGCCTTAAATCAAGATTAGTTGCAGTTTTGTCGTCTTTCCAGACGTT			
TTCTAAGAACGCGAGGCGTTTTAATTAAACCAAGTACCGCACTCATC			
CGCGCCCAATAGCAAGCAAATCAACCGATTGAGGGAGGGAAGGTA			
GAGAACAAGCAAGCCGTTTTTATAACCAATCAATAATCGGCTGTCTT			
TCCTTATCATTCCAAGAACGGGTATAGTTGCGCCGACAATGACAACAACC			
ATCCTAATTTACGAGCATGTAGAGCCAGCAAAATCACCAGTAGC			
CTGTTTATCAACAATAGATAAGTCCATTAAACGGGTAAAATACGTAATGC			
TCCAGACGACGACAATAAACAACGGCTTAATTGAGAATCGCCATATT			
GTAATAAGAGAATATAAAGTAACCTGTCGTGCCAGCTGCATT			
TAACAACGCCAACATGTAATTTATAAGAATAAACACCGGAATCATAA			
AGTATAAAGCCAACGCTCAACAGTAGGGAAACGTCACCAATGAAACCATC			
TTACTAGAAAAAGCCTGTTTAGTACTTTTTCAAATATATTTTAGTTA	yes		
TGTGATAAATAAGGCGTTAAAGGAGAGGCGGTTTGCGTATTGGGC			
ATTTCATCTTCTGACCTAAATTTGTCTGAGAGACTACCTTTTTAACC			
AATCGCAAGACAAAGAACGCGAGAAATCGGCATTTTCGGTCATAGCCCCC	yes		
TCCGGCTTAGGTTGGGTTATATATTAATTTTCCCTTAGAATCCTTGA			
GTGAATTTATCAAAATCATAGAGAGTTGCAGCAAGCGGTCCACGC			
AAACATAGCGATAGCTTAGATTACATTTAACAATTTCATTTGAATTA			
TTGCTTCTGTAAATCGTCGCTATTAATCAGAGCCGCCACCCTCAGAACCG			
CCTTTTTTAATGGAAACAGTACAAGCAAAAGAAGATGATGAAACAAA			
CATCAAGAAAACAAAATTAATTAAAAGAATAGCCCGAGATAGGG	yes		
GAATTATTCATTTCAATTACCTGATTGCGTAGATTTTCAGGTTTAAC			
CGGATTCGCCTGATTGCTTTGAATACGGGAGCCCCCGATTTAGAGCTTGA			
GTCAGATGAATATACAGTAACAGTCCTGATTGTTTGGATTATACTTC	yes		
ACGTAAAACAGAAATAAAGAAAGGTTGAGGCAGGTCAGACGA			
TGAATAATGGAAGGGTTAGAACCAACAGTTAATGCCCCCTGCCT			
ATGATGGCAATTCATCAATATAACAAGTGTAGCGGTCACGCTGCGCGTAA	yes		
GAAACCACCAGAAGGAGCGGAATGAGGATTTAGAAGTATTAGACTTT		yes (center)	yes (edge)
ATTAATTTTAAAAGTTTGAGTTGATAGCCCTAAAACATCGCC			
ACAAACAATTCGACAACTCGTATTGGCAAATCAACAGTTGAAAGGAA	yes		
TAGAGCCGTCAATAGATAATACATTTAAACATGAAAGTATTAAGAGGCTG			
TTGAGGAAGGTTATCTAAAATATTAGGAACCCATGTACCGTAACACTGAG			
CCCTCAATCAATATCTGGTCAGTACCAGCAGAAGATAAAACAGAGGT	yes		
CAGCAAATGAAAAATCTAAAGCATCATGGCTCATTATACCAGTCAGGACG			
GAGGCGGTCAGTATTAACACCGCGGCACAGACAATATTTTTGAATGG			
ATTAAAAATACCGAACGAACCTAAATCCTTTGCCCGAACGTT			
CTATTAGTCTTTAATGCGCGAACAAAGGGATTTTAGACAGGAACGGTACG			
GACCTGAAAGCGTAAGAATACGTAGTTGAGATTTAGGAATACCA			
ACCAGTAATAAAAGGGACATTCTACAATATTACCGCCAGCCATTGCA			
CGTCTGAAATGGATTATTTACGCGTCCAATACTGCGGAATCG			
ACAGGAAAAACGCTCATGGAAATTTGCGGATGGCTTAGAGCTTAATTGCT			
CGGCCTTGCTGGTAATATCCAGAATCAGTGAGGCCACCGAGTAAAAG			
GATTAGTAATAACATCACTTGGTTTTTTGGGGTCGAGGTGCCGTA			
AGTCTGTCCATCACGCAAATTAATGCTTTCCTCGTTAGAATCAGAGC			
CCAGAATCCTGAGAAGTGTTTTTATAGGCCAACAGAGATAGAACCCTTCT			

GGGAGCTAAACAGGAGGCCGATTCCGCTACAGGGCGCGTACTATGGT			
TGCTTTGACGAGCACGTATAACGGAGAAAGGAAGGGAAGAAAGCGAA			
CCACCACACCCGCCGCGCTTAATGCGAACATTATCATTTTGCGGAACAAA			
AGGAGCGGGCGCTAGGGCGCTGGTACCTTTTACATCGGGAGAAACAATAA			
CGGGGAAAGCCGGCGAACGTGGCCCGTTGTAGCAATACTTCTTT		yes (center)	yes (edge)
AAGCACTAAATCGGAACCCTAAAAACAAGAGTCCACTATTAAAGAAC			
ACTACGTGAACCATCACCCAAATCAATTTAAATATGCAACTAAAGTACGG			
GTGGACTCCAACGTCAAAGGGCGTCCTGTTTGATGGTGGTTCCGAAA			
TTGAGTGTTGTTCCAGTTTGGCAAGTTACAAAATCGCGCAGAGGC			
TCGGCAAAATCCCTTATAAATCAAGACGCTGAGAAGAGTCAATA			
TGGTTTGCCCCAGCAGGCGAAAATAGATACATTTCGCAAATGGTCAATAA			
GCCCTTCACCGCCTGGCCCTGAGAATGGTTTGAAATACCGACCG			
GCCAGGGTGGTTTTTCTTTTCACACTCACATTAATTGCGTTGCGCTC	yes		
AATGAATCGGCCAACGCGCGGGGCAGAGGCATTTTCGAGCCA		yes (center)	yes (edge)
ACTGCCCGCTTTCCAGTCGGGAACTGATAAATTGTGTCGAAATCCGCGAC			
TGGGGTGCCTAATGAGTGAGCTAGCGCGAGCTGAAAAGGTGGCATCA	yes		
ACAATTCCACACAACATACGACAAAAAGATTAAGAGGAAGCCCGA		yes (center)	
ATTCTACTAATAGTAGTAGCATTAACAGTTGATTCCCAATTCTGCGA			
CCTGTTTAGCTATATTTTCATTTGGGCAGTGAGACGGGCAACAGCTGATT	yes		
ACGAGTAGATTTAGTTTGACCATAAAAACCGTCTATCAGGGCGATGGCCC			
TGTCTGGAAGTTTCATTCCATATAGGATTAGAGAGTACCTTTAATTG			
GAATATAATGCTGTAGCTCAACATGTCCTGAGTAGAAGAACTCAAACTAT			
CTCCTTTTGATAAGAGGTCATTTATTCGAGCTTCAAAGCGAACCAGA			
CCGGAAGCAAACTCCAACAGGTCGTGTGAAATTGTTATCCGCTC			
AAGACTTCAAATATCGCGTTTTACCCTCAAATGCTTTAAACAGTTCA	yes		
ATAGTCAGAAGCAAAGCGGATTGCATAGGCGCAGACGGTCAATCATAAGG			
GAAAACGAGAATGACCATAAATCAAGAAGTTTTGCCAGAGGGGGTAA			
TCATAAATATTCATTGAATCCACCTACATTTTGACGCTCAAT			
TAGTAAAATGTTTAGACTGGATATAACGCCAAAAGGAATTACGAGGC			
ACCAAAATAGCGAGAGGCTTTTGCAACTTCATCAAGAGTAATCTTGACAA		yes (center)	
ATAGTAAGAGCAACACTATCATATAAAACGAACTAACGGAACAACAT	yes		
CATTCAACTAATGCAGATACAATTGGCAGATTCACCAGTCACACG			
TATTACAGGTAGAAAGATTCATCCTGCAACAGTGCCACGCTGAGAGCCAG			
TTGGGAAGAAAAATCTACGTTAAAGAAACACCAGAACGAGTAGTAAA			
ACCTTATGCGATTTTAAGAACCCTGTAGCATTCCACAGACAGCCC			
TTGGGCTTGAGATGGTTTAATTTCTCCAAAAAAAAAGGCTCCAAA			
AGTGAATAAGGCTTGCCCTGACGACCCTCGTTTACCAGACGACGATAAAA			
GAACCGGATATTCATTACCCAAACACCCTCAGCAGCGAAAGACA	yes		
ACCAGGCGCATAGGCTGGCTGACAAAAATCAGGTCTTTACCCTGACTATT			
GAACCGAACTGACCAACTTTGAAATTATACCAAGCGCGAAACAAAGT			
CTGCTCCATGTTACTTAGCCGGAACGGCCGGAAGCATAAAGTGTAAAGCC			
ACAACGGAGATTTGTATCATCGCAAAGAGGCAAAAGAATACACTAAA	yes		
ACACTCATCTTTGACCCCCAGCGCGGCTACAGAGGCTTTGAGGACTA			
CACTACGAAGGCACCAACCTAAAACGCCGACAAAAGGTAAAGTAATTCTG			
AAGACTTTTTCATGAGGAAGTTTGGTCGCTGAGGCTTGCAGGGAGTT			
GCATCGGAACGAGGGTAGCAAAGAGGACAGATGAACGGTGTACAG			
AAAGGCCGCTTTTGCGGGATCGTTTTATCAGCTTGCTTTCGAGGTGA			
ATCGCCCACGCATAACCGATATATTCCCTGAACAAGAAAAATAATATCCC			
ATTTCTTAAACAGCTTGATACCGGGAACAACTAAAGGAATTGCGAAT			
AGGAGCCTTTAATTGTATCGGTCAACGTAACAAAGCTGCTCATTC			
AATAATTTTTTCACGTTGAAAATATTTTGCTAAACAACTTTCAACAG			
TTTCAGCGGAGTGAGAATAGAAAGCGAACCTCCCGACTTGCGGGAGGTTT			
AGTAAATGAATTTTCTGTATGGGCAACTTTAATCATTGTGAATT			
TCATAGTTAGCGTAACGATCTAAGCCACCCTCAGAGCCACCACCCTC			
TTTCGTCACCAGTACAAACTACAACGCCTTGCTGAACCTCAAATATCAAA			
ATTTTCAGGGATAGCAAGCCCAAGGGTTGATATAAGTATAGCCCGGA			
AGAACCGCCACCCTCAGAACCTATTTTGCACCCAGCTACAATTTT			
ATAGGTGTATCACCGTACTCAGGGGAAGCGCATTAGACGGGAGA			
CAGGCGGATAAGTGCCGTCGAGACTTTAGGAGCACTAACAACTAATAGAT			
AGACTCCTCAAGAGAAGGATTAGTAATAAGTTTTAACGGGGTCAGTG			
ATTTCGGAACCTATTATTCTGTATCATCATATTCCTGATTATCAG		yes (center)	
CCTTGAGTAACAGTGCCCGTATAAAATAAATCCTCATTAAAGCCAGA			
CTTTTGATGATACAGGAGTGTACTGGAAGTCAGAGGGTAATTGAGCGCTA			
ATGGAAAGCGCAGTCTCTGAATTGAGCCGCCACCAGAACCACCACCA			
TTGGCCTTGATATTCACAAACTACCATATCAAAATTATTTGC			
GAGCCGCCGCCAGCATTGACAGGTAAATCAATATATGTGAGTGAATAACC		yes (center)	

Table S3. List of DNA oligos for DNA nanorod

**DNA nanorod sequence**	biotin sites	10 binding sites	4 edge binding sites
TCAGGCTGCGCAACCTAGGGCGCTGGCAATCGTCTGAAATGG			
CATAACGCCAAAAGTTGCTAAACAACTTCCAATAGGAACCCA			
CCGCTTCTGGTGCCCCACACCCGCCGCGACAGGAAAAACGCT			
TATCGGCCTCAGGAATGGTTGCTTTGACTTGCTGGTAATATC			
CATCGTAACCGTGCGAATCAGAGCGGGAATAACATCACTTGC			
GATTGACCGTAATGTTAGACAGGAACGGTCACGCAAATTAAC			
TCAGTTGAGATTTAAAGGAACAACTAAACCACCCTCAGAGCC			
AACGAACTAACGGATGAAAATCTCCAAAGGTTTAGTACCGCC			
TATACCAGTCAGGAGTATCGGTTTATCAATATAAGTATAGCC			
ATCATTGTGAATTAAGCTTGATACCGATTTTTGCTCAGTACC			
CGAGTAGTAAATTGGCCCACGCATAACCAGAGGCTGAGACTC			
TCATTCAGTGAATAGAGTTAAAGGCCGCTGCCTATTTCGGAA			
AGAACCGGATATTCAAAGACAGCATCGGGTGCCTTGAGTAAC			
GGCGCATAGGCTGGTTGAGGACTAAAGAGATGATACAGGAGT			
TGACCAACTTTGAAGGGTAAAATACGTATCTCTGAATTTACC			
GCCGGAACGAGGCGCGAAAGAGGCAAAACAAACAAATAAATC			
GATAAATTGTGTCGCCCAGCGATTATACGAAGTATGTTGAGG			
TTGCGTATTGGGCGCTTTTCACCAGTGAAATAGATTAGAGCC			
TATCATAACCCTCGCGTCTTTCCAGACGGTACAAACTACAAC			
CAGCTGCATTAATGGCCTGGCCCTGAGATGAGGAAGGTTATC			
TTGCGCTCACTGCCTGCCCCAGCAGGCGATCAATATCTGGTC			
GCCTGGGGTGCCTATCGGCAAAATCCCTTCTAAAGCATCACC			
ACAATTCCACACAAGGTTGAGTGTTGTTCCTGCAACAGTGCC			
TCATGGTCATAGCTAGAACGTGGACTCCGCAGAAGATAAAAC			
TCGACTCTAGAGGAAGGGCGATGGCCCAAGCCCTAAAACATC			
GTTGTAAAACGACGTTTTGGGGTCGAGGAATATTTTTGAATG			
GGCGATTAAGTTGGAAGGGAGCCCCCGAGAACCCTTCTGACC			
CTTCGCTATTACGCACGTGGCGAGAAAGACACGACCAGTAAT			
GAGAAGTGTTTTTAGTCGGATTCTCCGTAAATGTGAGCGAGT			yes (edge)
ATTATTTACATTGGAATTAATTACATTTCGTTATACAAATTC			
CATGGAAATACCTAATGGAAACAGTACACGGAATCATAATTA			
CAGAACAATATTACTGCTTCTGTAAATCACCGACCGTGTGAT			
CTGAGTAGAAGAACTCCTTGAAAACATATAGTTAATTTCATC			
CGTTGTAGCAATACGAGTCAATAGTGAATCGCAAGACAAAGA			
GAGGCCACCGAGTAACCTTTTTAACCTCGTTGGGTTATATAA			yes (edge)
ACCACCCTCATTTTCAAAGACAAAAGGGAACAAAGTTACCAG			
ACCCTCAGAACCGCGTAAATATTGACGGATCTTACCGAAGCC			
CGGAATAGGTGTATCGTCACCGACTTGAAGCCCAATAATAAG			
AGGCGGATAAGTGCCACCAGTAGCACCATGAGCGCTAATATC			
CTCAAGAGAAGGATCAATGAAACCATCGGGGAGAATTAACTG			
CCTATTATTCTGAAAATCAAGTTTGCCTTTTACAGAGAGAAT			
AGTGCCCGTATAAACGGCATTTTCGGTCGAAACGATTTTTTG			
GTACTGGTAATAAGTTTCATAATCAAAATTACAAAATAAACA			
GTTCCAGTAAGCGTCGCCTCCCTCAGAGTACCAACGCTAACG			
GCCTGTAGCATTCCCAACATATAAAAGAGCAGTATGTTAGCA			
CTCATTAAAGCCAGAGCCACCACCCTCATAGTTGCTATTTTG			
CAGGTCAGACGATTCGCCGCCAGCATTGACCTCCCGACTTGC			
GTCAATAGATAATACAACTCGTATTAAAAAGGCTTATCCGGT			
TAAAATATCTTTAGAAGTTTGAGTAACAAGGAATCATTACCG			
AGTTGGCAAATCAACAGAAGGAGCGGAACGCACTCATCGAGA			
TTGCTGAACCTCAAATGGCAATTCATCAGTCTTTCCTTATCA			
ACGCTGAGAGCCAGTCTGAATAATGGAAATCCTAATTTACGA			
AGAGGTGAGGCGGTTTGCACGTAAAACATATCAACAATAGAT			
GCCATTAAAAATACGTTTAACGTCAGATGACAATAAACAACA			
GCTATTAGTCTTTACGGGAGAAACAATAATAAAGTACCGACA			
TGTACCGTAACACTTTTTGTCACAATCAGGAATACCCAAAAG			
TGAAAGCGTAAGAAAAGTTACAAAATCGGTAATTTAGGCAGA			

AAAAGGGACATTCTCCTGAGCAAAAGAATAGGGCTTAATTGA			
TTACCAGTATAAAGCGGTAATCGTAAAAATCGGTGCGGGCCT			
CTAGAAAAAGCCTGTGATAATCAGAAAAGCGCCATTCGCCAT			
AAATAAGGCGTTAAAAATATTTAAATTGCCAGCTTTCCGGCA			
TTCTGACCTAAATTATTAAATTTTTGTTGGGGACGACGACAG			
ACGCGAGAAAACTTACGCCATCAAAAATGGTGTAGATGGGCG			
AAGGAAACCGAGGACGTCATAAATATTCAACTAATGCAGATA			
CTATATGTAAATGCCTTTCATCAACATTGGGAACAAACGGCG			
CTTTTTAAGAAAAGGTTCAGAAAACGAGGGTAGAAAGATTCA			
AGCAAGAAACAATGTACCCTGACTATTAATCTACGTTAATAA			
AGAGAGATAACCCAAAAGATTAAGAGGAAAGAACTGGCTCAT			
AACACCCTGAACAATAATTCGAGCTTCATAATTTCAACTTTA			
AACATAAAAACAGGACAGGTCAGGATTAGAGAAACACCAGAA			
TTTAACGTCAAAAAAAGAGGTCATTTTTCGTAACAAAGCTGC			
GCCATATTATTTATTATAATGCTGTAGCGAGTAATCTTGACA			
AGCGTCTTTCCAGATACGGTGTCTGGAACGGTGTACAGACCA			
CACCCAGCTACAATAATTCTGCGAACGATAAGGGAACCGAAC			
GGGAGGTTTTGAAGTTCGCAAATGGTCACTCCATGTTACTTA			
ATTCTAAGAACGCGGGGCGCGAGCTGAATTGTATCATCGCCT			
CGCCCAATAGCAAGTAGCATTACATCCACGGGGAGAGGCGGT			
ACAAGCAAGCCGTTAGCAAAATTAAGCAGAAACCTGTCGTGC			
TTCCAAGAACGGGTGGTTGTACCAAAAATCACATTAATTGCG			
GCATGTAGAAACCAAGAAGCCTTTATTTGCATAAAGTGTAAA			
AACTGGCATGATTATAGTAAAATGTTTAAGTAAGAGCAACAC			
AAGTCCTGAACAAGCTCATATATTTTAAATTGTTATCCGCTC			
TGTTCAGCTAATGCAGATTCAAAAGGGTGCTCGAATTCGTAA			
AAAGGTAAAGTAATATCAATATGATATTTGCATGCCTGCAGG			
GGCATTTTCGAGCCGGAGAGGGTAGCTATTCCCAGTCACGAC			
GAATCGCCATATTTGTCATTGCCTGAGAGGGATGTGCTGCAA			
CTGAGGCTTGCAGGAGGCTTGCCCTGACGAGAGTACCTTTAA			
AAATCGGAACCCTAGTAACGCCAGGGTTTTTTTGAGAGATCT			
ATATGTACCCCGGTTTTAGTATCATATGAACAATTTCATTTG			
AAGATTGTATAAGCATAAGAATAAACACTAAATCAATATATG			
TTGTTAAAATTCGCTAATGGTTTGAAATGTCGCTATTAATTA			
TTTAACCAATAGGATTTCAAATATATTTGCGATAGCTTAGAT			
CTTCCTGTAGCCAGTGATGCAAATCCAATTTATCAAAATCAT			
AAATGCTTTAAACATAAGCAGATAGCCGCGACATTCAACCGA			
AAAAATCAGGTCTTAAATAGCAATAGCTAAATTATTCATTAA			
GCGGATTGCATCAACAAGAATTGAGTTAGCCATTTGGGAATT			
GGAAGCAAACTCCAGAAGCGCATTAGACATAGCAGCACCGTA			
TTGCTCCTTTTGATTGAAAATAGCAGCCTTAGCGTCAGACTG			
GCTTAATTGCTGAACCCAATCCAAATAAATAGCCCCCTTATT			
ATATGCAACTAAAGGCCTAATTTGCCAGTCACCGGAACCAGA			
AACAGTTGATTCCCTTTATCCTGAATCTCCGCCACCCTCAGA			
GCCAGAGGGGGTAAAGACTCCTTATTACAACGCAAAGACACC			
TATATTTTCATTTGAGGCGTTTTAGCGAACAGGAGTAGACTT			
TCTACTAATAGTAGCAAATCAGATATAGTCCTTTGCCCGAAC			
CAAGGCAAAGAATTTTTATTTTCATCGTTTATCATTTTGCGG			
CATAAAGCTAAATCATTAAACCAAGTACTTATCATCATATTC			
AATACTTTTGCGGGATCAATAATCGGCTATATAATCCTGATT			
TAATGTGTAGGTAAAGAACGCGCCTGTTGAAATAAAGAAATT			
ACAGTCAAATCACCTCTGTCCAGACGACGAATATACAGTAAC			
GATAAATTAATGCCAGTAATAAGAGAATACGGATTCGCCTGA			
CAATACTGCGGAATAACGCAATAATAACATAGAAAATTCATA			
ACAAAGGCTATCAGAACAACGCCAACATCGCAGAGGCGAATT			
AGAGAATCGATGAACCAACGCTCAACAGGATGATGAAACAAA			
GAAAGGAGCGGGCGTGTTGGGAAGGGCGCTAGCATGTCAATC			
ACAGGGCGCGTACTAGATCGCACTCCAGTAAACGTTAATATT			
CGATTAAAGGGATTGGATAGGTCACGTTAATTCGCGTCTGGC			

TAATTTTTTCACGTACAACATTATTACAAATGACCATAAATC			
TGAATTTCTTAAACCCTTATGCGATTTTAGCCCGAAAGACTT			
CGGCTACAGAGGCTCTGACCTTCATCAATCAACATGTTTTAA			
GCACCAACCTAAAACAGACGGTCAATCAGTAGATTTAGTTTG			
AGTACAAGGTTTTTCCAGGGTCGGAGATAAGGTGGCATCAAT			
GGTCCACGCTGGTTCGCTTTCCAGTCGGATAAAGCCTCAGAG			
ATAGCCCGAGATAGCATACGAGCCGGAACAACGCAAGGATAA			
TTCTGTATGGGATTGAATTACGAGGCATGACTGGATAGCGTC			
AAAAACCGTCTATCTCCCCGGGTACCGAGAGAAAGGCCGGAG			
AGCGAGAGGCTTTTATAAAAACCAAAAT			
TAGTTAGCGTAACGACAGACAGCCCTCA			yes (edge)
ATACATAAAGGTGGAACGTAGAAAATAC			yes (edge)
CAAATATCGCGTTTAGTCAGAGGGTAATTTACCATTAGCAAG			
ACCATTAGATACATCCTTAAATCAAGATGAGCCGCCACCAGA			
AAATTTTTAGAACCAAAAATAATATCCCGGGTTAGAACCTAC			
GCTGCGCGTAACCAGGAAACCAGGCAAAGCCCCAAAAACAGG	yes		
TGCTTTCCTCGTTAATCTGCCAGTTTGAAAATCAGCTCATTT	yes		
GGAGTGAGAATAGAGGAATACCACATTCATTGAATCCCCCTC	yes		
AGGAGCCTTTAATTCGTTGGGAAGAAAATAGTCAGAAGCAAA	yes		
TGACAACAACCATCGGCTTGAGATGGTTAAGCGAACCAGACC	yes		
ATCTAAAGTTTTGTTTTACCAGACGACGGCAAAAGAAGTTTT	yes		
CACCCTCAGCAGCGATTACCCAAATCAAGCGGATGGCTTAGA	yes		
AGTTTCCATTAAACAGAGGACAGATGAAGTTTCATTCCATAT	yes		
ACTCATCTTTGACCAAATCCGCGACCTGATAACCTGTTTAGC	yes		
GATTGCCCTTCACCAATCGGCCAACGCGATAAATCATACAGG	yes		
TGGTGGTTCCGAAAATGAGTGAGCTAACCATTATGACCCTGT	yes		
GAGTCCACTATTAAGTTTCCTGTGTGAAATGCAATGCCTGAG	yes		
ACCCAAATCAAGTTGCCAGTGCCAAGCTCAACCGTTCTAGCT	yes		
GGGAAAGCCGGCGACAGCTGGCGAAAGGGTCTGGAGCAAAC	yes		
ACGGAATAAGTTTAGAGTTTCGTCACCATTAGTAAATGAATT			
TGGTTTACCAGCGCCAGGGATAGCAAGCTCAACAGTTTCAGC			
TTGAGGGAGGGAAGCACCCTCAGAACCGGGAATTGCGAATAA			
AGGTGAATTATCACCACCGTACTCAGGAAAAAAGGCTCCAAA			
AGAGCCAGCAAAATCGTCGAGAGGGTTGGCTTGCTTTCGAGG		yes (center)	
GCCGGAAACGTCACTAGGATTAGCGGGGAGTTGCGCCGACAA			
ATCAGTAGCGACAGACATGAAAGTATTAGATATATTCGGTCG		yes (center)	
TAGCGCGTTTTCATCAGTTAATGCCCCCTTTTGCGGGATCGT			
AGCGTTTGCCATCTTTTTAACGGGGTCAAACGAGGGTAGCAA		yes (center)	
GCCACCACCGGAACCATACATGGCTTTTCTTTTTCATGAGGA			
ACCGCCACCCTCAGAATGGAAAGCGCAGATGCCACTACGAAG		yes (center)	
ACCACCACCAGAGCGGCCTTGATATTCAGAATACACTAAAAC			
TACAAACAATTCGACATTTGAGGATTTACAAGCGCGAAACAA		yes (center)	
GTTATTAATTTTAAGAGCACTAACAACTGACGGGCAACAGCT			
AACAAAGAAACCACCAGTTGAAAGGAATGAGTTGCAGCAAGC		yes (center)	
CTGATTATCAGATGATATCAAACCCTCAAAAATCCTGTTTGA			
GTTTGGATTATACTCAGCAAATGAAAAATATAAATCAAAAGA		yes (center)	
CATATCAAAATTATCAGTATTAACACCGCCAGTTTGGAACAA			
GCGTAGATTTTCAGCGAACGAACCACCAAACGTCAAAGGGCG		yes (center)	
AGTACCTTTTACATATGCGCGAACTGATCTACGTGAACCATC			
TTGCTTTGAATACCTACGTGGCACAGACTGCCGTAAAGCACT			
ATTCATTTCAATTAGGCCAACAGAGATATTTAGAGCTTGACG			
CATCAAGAAAACAACAGATTCACCAGTCGAAGGGAAGAAAGC			
AATTACCTTTTTTACATTTTGACGCTCAAGTGTAGCGGTCAC			
TGAGTGAATAACCTCGCCAGCCATTGCACTTAATGCGCCGCT		yes (center)	
ATTTTCCCTTAGAATCAAACTATCGGCCGAGCACGTATAACG			
TAAGACGCTGAGAATTCTTTGATTAGTAGCTAAACAGGAGGC		yes (center)	
AGGTCTGAGAGACTAAAGAGTCTGTCCATACGCCAGAATCCT			

Table S4. List of DNA oligos for DNA nanotiles

**DNA nanotile sequence**	biotin sites	10 binding sites	4 edge binding sites
TCAGGAGGTTTAGTACCGCCACCCTCAGAAC			
TTTGCTAAGTAAATGAATTTTCTGAGTGCCTT			
GAGTAACAGTTTTAACGGGGTCTATGGGAT			
TAACCGATATGACAACAACCATCGCACCACCC			
TCAGAGCCGCCACCCTCAGAGCCCCACGCA			
CACTAAAATAAAACGAAAGAGGCAACGTCACC			
AATGAAATTAGCAAGGCCGGAAAAAGAATA			
TGACCTTCTACAGACCAGGCGCATACCACGGA			
ATAAGTTAAGAAACGCAAAGACAGGCTGGC			
AAAATCTAATACCAGTCAGGACGTGCAAGAAA			
CAATGAAAGCCCAATAATAAGATGGGAAGA			
GGGGTAATTTTGCAAAAGAAGTTTAAATAAAC			
AGCCATAAATTTGCCAGTTACATGCCAGAG			
TTCGAGCTAAGACTTCAAATATCGATCGTAGG			
AATCATTGCCGTTTTTATTTTCCGTTTTAA			
AGTAGATTAGTTGATTCCCAATTCTTCTGTCC			
AGACGACCAAAAGGTAAAGTAATGCGAACG			
CCCTGTAATCGGTTGTACCAAAAATAAGGCGT			
TAAATAACGACCGTGTGATAAACATTATGA			
AGGCTATCTAGCTATTTTTGAGAGAAGACGCT			
GAGAAGAGCGATAGCTTAGATTATCTACAA			
ACGCCATCCAGCTCATTTTTTAACAGGCGAAT			
TATTCATTACAAAATCGCGCAGCAATAGGA			
AGCTTTCCCCTCAGGAAGATCGCACATCAATA			
TAATCCTCAGATGATGGCAATTCTCCAGCC			
GGTCGACTCAGTGCCAAGCTTGCAGGAGCACT			
AACAACTTCTAAAATATCTTTATGCCTGCA			
TTAATGAAGGGAAACCTGTCGTGCATTAAAAA			
TACCGAACCCTAAAACATCGCCCAGCTGCA			
CCCGAGATATCCCTTATAAATCAAAAACGCTC			
ATGGAAACCATTGCAACAGGAAAAGAATAG			
GAGCTTGACGGGGAAAAAAGGGATTTTAGAC			
TAGGAACCCATGTACACAACGCC			
TGTAGCATAATTTTTTCACGTTCCTTTAAT			
TGTATCGAGCGAAAGACAGCATTGAGGACT			
AAAGACTCATCGCCTGATAAATTTAGCCGG			
AACGAGGATTCAGTGAATAAGGTAAATTGG			
GCTTGAGTAGGAATACCACATTTTACGAGG			
CATAGTACCCCTCAAATGCTTTCAAAAATC			
AGGTCTTTCCTTTTGATAAGAGCTGAATAT			
AATGCTGGGGCGCGAGCTGAAATTAACATC			
CAATAAATTTAAATGCAATGCCGAGAAAGG			
CCGGAGAATGTCAATCATATGTCAGGAAGA			
TTGTATAACAACCCGTCGGATTTGGGATAG			
GTCACGTTGGGAAGGGCGATCGGAAAGGGG			
GATGTGCAATTGTTATCCGCTCAAAGTGTA			
AAGCCTGAGTGAGACGGGCAACGAGTTGCA			
GCAAGCGCAAAGGGCGAAAAACCATCACCCAAATCAA			yes (edge)
GCGAATAATTCCACAGACAGCCCTGGGATAGCAAGCCCAA			yes (edge)
CCCTCAGCGTTTATCAGCTTGCTTAAGGAATT	yes		
ATTTGTATTTTTCATGAGGAAGTTGATCGTCA			
AGCTGCTCCGCAGACGGTCAATCACAACGGAG	yes		
TTGAGATTATGGTTTAATTTCAACCGTAACAA			
ATTGAATCAGAGCAACACTATCATTTCATCAG	yes		
TTAATTGCTACCCTGACTATTATAAAATATTC			
TTCATTTGTAGCTCAACATGTTTTGAGTACCT	yes		
TCATATATTCATACAGGCAAGGCAGCTATATT			
AAACTAGCCAGTCAAATCACCATCTAGAACCC	yes		
AGCGAGTAAGCAAATATTTAAATTTAATCGTA			
GCAACTGTTGGTGTAGATGGGCGCTAAATGTG	yes		
CTGTGTGATGCAAGGCGATTAAGTCAGGCTGC			
TTTTCACCGGGTGCCTAATGAGTGGCTGTTTC	yes		
TCCAACGTGTCCACGCTGGTTTGCGGTTTTTC			
GTTTTTTGGGGTCGAGACGTGGAC			
ACCACCCTCATTTTCACATAGTTAGCGTAACGTAGAAAGG			

AACAACTATCGAGGTGAATTTCTTAAGGCCGC		yes (center)	
TTTTGCGGTCCATTAAACGGGTAAAGCGCGAA			
ACAAAGTATAAGGGAACCGAACTGTCATTACC			
CAAATCAATTTAATCATTGTGAATATTACAGG			
TAGAAAGAAACCCTCGTTTACCAGACTGCGGA			
ATCGTCATGTCAGAAGCAAAGCGGACAGGTCA			
GGATTAGAAAATATGCAACTAAAGGTCAATAA		yes (center)	
CCTGTTTAAAGAATTAGCAAAATTCAAGGATA			
AAAATTTTAATATGATATTCAACCGAGAATCG			
ATGAACGGGTAAACGTTAATATTTCAGCTTTC			
ATCAACATATCGTAACCGTGCATCAGCGCCAT			
TCGCCATTTGGGTAACGCCAGGGTCGTAATCA			
TGGTCATAAGCTAACTCACATTAATATTGGGC		yes (center)	
GCCAGGGTCCCAGCAGGCGAAAATAGTCCACT			
ATTAAAGAGTGCCGTAAAGCACTA			
AGTGAGAAATCTAAAGTTTTGTCGCCACCCTCAGAGCC			
GGGAGTTAAAACAGCTTGATACCTCAGCGG			
TTATACCAAATACGTAATGCCACGCTTGCA			
CCGGATATACCAACTTTGAAAGACCAGCGA			
ACAACATTTACCTTATGCGATTTACAAGAA			
CGTCCAATACGACGATAAAAACCTAACGGA			
AAACTCCAATTGCATCAAAAAGATGGATAG			
CGCAAATGTACGGTGTCTGGAAGCGGAAGC			
TTTCAACGAAGCAATAAAGCCTCTACATTT			
GCAAACAAGTTCTAGCTGATAAAGCCTTTA			
CCTGTAGCTGTTAAAATTCGCATGTCTGGA			
CCAGGCAATGCCAGTTTGAGGGGTGGCCTT			
CTCGAATTTTTCCCAGTCACGACCCGGAAA			
GGTTTGCGTTGCGTTGCGCTCACTACCGAG			
GGAACAAGCCTGTTTGATGGTGGGAGAGGC			
AATCGGAACCCTAAACCAGTTT			
CGCCACCCTCAGAACCGTCTTTCCAGACGTTAACAACTTT			
CAACAGTTGATAGTTGCGCCGACAATATTCGG			
TCGCTGAGTACGAAGGCACCAACCCACTCATC			
TTTGACCCGGACAGATGAACGGTGATCAAGAG			
TAATCTTGTAAGAACTGGCTCATTCGTTAATA			
AAACGAACAAAATAGCGAGAGGCTAGTAAAAT			
GTTTAGACTTAAGAGGAAGCCCGATCAAAGCG		yes (center)	
AACCAGACTTTCATTCCATATAACTAGTTTGA			
CCATTAGAAGAGCATAAAGCTAAATACTTTTG			
CGGGAGAATTAATGCCGGAGAGGGAGGTCATT			
GCCTGAGATAAATTTTTGTTAAATAAAAATAA		yes (center)	
TTCGCGTCACGACGACAGTATCGGGGCACCGC			
TTCTGGTGGTTGTAAAACGACGGCCTAGAGGA			
TCCCCGGGTGCCCGCTTTCCAGTCTCGGCCAA			
CGCGCGGGTTCCGAAATCGGCAAAAGGGTTGA			
GTGTTGTTGGGAGCCCCCGATTTA			
TGGTAATAAGTGCCCGTATAAACAAGGTGTATCACCGTAC			
CTCAGAACCGCCACCAGAACCACCGAGTGTAC			
CATTACCACCATCGATAGCAGCACCCGCCACC	yes		
ACATATAATATTTTGTCACAATCAAGTAGCAC			
TTGAGTTAATAGCAATAGCTATCTAGGTGGCA	yes		
CAGAGCCTTTATTTATCCCAATCCCACAAGAA			
ACAAGCAAACCGCGCCCAATAGCACGTCTTTC	yes		
AGTACCGAGACAATAAACAACATGCATCGAGA			
TGAAATACGAATAAACACCGGAATGAATATAA	yes		
AAAACATAGTCAATAGTGAATTTATAATGGTT			
TACCAAGTTTCAATTACCTGAGCAAATCCTTG	yes		
CTGATTATGATTGTTTGGATTATAGCTTTGAA			
GAAGGTTAAATAGATTAGAGCCGTTCATATTC	yes		
ACTGATAGCGAACCACCAGCAGAAGAATTGAG			
ACCGCCAGTACCTACATTTTGACGATGCGCGA	yes		
AGGAACGGTACGCCAGACAATATT			
AAGTATAGCCCGGAATGTTAATGCCCCCTGCCGCTTTTGA			
TGATACAGACCAGAGCCGCCGCCAACCGCCTC			
CCTCAGAGCGTAATCAGTAGCGACAGCCAGCA			
AAATCACCATAGAAAATTCATATGGAAAATAC			
ATACATAATACCGAAGCCCTTTTTTATCAGAG			
AGATAACCAAATAAGAAACGATTTTACCAACG			

CTAACGAGAGCAAATCAGATATAGAACCAAGT		yes (center)	
ACCGCACTTTCAGCTAATGCAGAACGAGCCAG			
TAATAAGACATAATTACTAGAAAATCTTCTGA			
CCTAAATTTCAAAATCATAGGTCTATTAATTT			
TCCCTTAGAAAGAAGATGATGAAAACGGATTC		yes (center)	
GCCTGATTCTTCTGAATAATGGAAAGGAGCGG			
AATTATCACAATAGATAATACATTAATCAACA			
GTTGAAAGGATAAAACAGAGGTGATGGCTATT			
AGTCTTTACTCAATCGTCTGAAATGCTGGTAA			
TATCCAGAAATCCTGAGAAGTGTT			
CATACATGTATTTCGGAACCTATCGAGAGGGTTGATAT			
CCACCGGAGCATTGACAGGAGGTTAAGCGT			
GGAATTAGAGAATCAAGTTTGCCAGAGCCA			
CAAACGTAGTTTACCAGCGCCAACCATTTG			
AGCGCTAAAAGAAAAGTAAGCAGATGTTAG			
CTGAATCTTTTGTTTAACGTCAAGTAATTG			
GGGTATTAAAGGCTTATCCGGTATTTTATC			
GGCATTTTCGCGCCTGTTTATCACAAGAAC			
TAATTTCAAGCCTGTTTAGTATCAGGCAGA			
CGCTATTAGAGAGACTACCTTTTTTTTAGT			
AAACAATACAAACATCAAGAAAAAAATCGT			
CCACCAGAGGGTTAGAACCTACCTCGGGAG			
AGTTGGCATGAGGATTTAGAAGTAAAGAAA			
TTTTTGAAGGCGGTCAGTATTAATCTGGTC			
TCGGCCTTGGATTATTTACATTGGACAATA			
TTTATAATCAGTGAGCAAACTA			
GGCGGATAAGTGCCGTTATTCTGAAACATGAAGAATTTAC			
CGTTCCAGTGAGGCAGGTCAGACGCAAAATCA		yes (center)	
CCGGAACCTTTAGCGTCAGACTGTCCGTCACC			
GACTTGAGAGACAAAAGGGCGACACTCCTTAT			
TACGCAGTATAGCCGAACAAAGTTTGAACAAA			
GTCAGAGGAAATGAAAATAGCAGCTTGCACCC			
AGCTACAATTCTAAGAACGCGAGGTCTTTCCT			
TATCATTCACAATAGATAAGTCCTCGCCAACA		yes (center)	
TGTAATTTATATGCGTTATACAAAAACTTTTT			
CAAATATATAACCTCCGGCTTAGGATAACCTT			
GCTTCTGTCAAAATTAATTACATTAACAGTAC			
CTTTTACAATATCAAAATTATTTGTATCATTT			
TGCGGAACATTAGACTTTACAAACAAACCCTC			
AATCAATACACCGCCTGCAACAGTAAGAATAC		yes (center)	
GTGGCACAGCAGATTCACCAGTCACCTGAGTA			
GAAGAACTGCCACCGAGTAAAAGA			yes (edge)
CAGTCTCTAGTATTAAGAGGCTGAGTTTTGCTCAGTACCA			yes (edge)
TTCATAATATTGGCCTTGATATTCGGAAAGCG			
AATTATCAAGCGCGTTTTCATCGGGCCATCTT			
GATTAAGATTCAACCGATTGAGGGTAAAGGTG			
GAACACCCACCAGAAGGAAACCGAACTGGCAT			
TTGCTATTCTTTACAGAGAGAATAAATTAACT			
ATCGGCTGCGTTTTAGCGAACCTCAAGATTAG			
TTTAACAAGAACAAGAAAAATAATAATCAATA			
CGCGAGAATTCTTACCAGTATAAATCGCCATA			
GTGAGTGATTGGGTTATATAACTAACAAAGAA			
TATACAGTTAACAATTTCATTTGACAATATAT			
AGTAACATCACGTAAAACAGAAATCAGATGAA			
CAAATATCAATTCGACAACTCGTAAAAGTTTG			
GAAAGCGTGCCACGCTGAGAGCCACTGAACCT			
ATCACTTGCACGACCAGTAATAAATCTGACCT			
GTCTGTCCATCACGCAGTAATAAC			

Table S5. Calculation of energy needed for spontaneous wrapping of DNs. See Supplementary Figure. 9 for schematic illustrations. The adhesion energy provided by the interaction between DNs and cell membranes (the insertion of cholesterol into lipid membranes) is $n(k_B T)$. n is the number of cholesterol tags inserted into the membrane. κ is the bending modulus of the cellular membrane. In the nanotiles, the curvature radius for membrane bending is related to the tile thickness L_y.

<table>
<tr><th colspan="2">DNs</th><th>Membrane bending energy to wrap the DN</th></tr>
<tr><td rowspan="2">Nanosphere</td><td>Horizontal</td><td rowspan="2">$\frac{1}{2}\kappa(\frac{1}{R}+\frac{1}{R})^2 A_{sphere} = 8\pi\kappa$
R is the radius of sphere.</td></tr>
<tr><td>Vertical</td></tr>
<tr><td rowspan="2">Nanorod</td><td>Horizontal</td><td>$\frac{1}{2}\kappa(\frac{1}{R})^2 A_{rod,horizontal} = \frac{\kappa}{2R}\varphi L$
R is the radius of the rod. φ the portion of the rod that has been engulfed.</td></tr>
<tr><td>Vertical</td><td>$\frac{1}{2}\kappa(\frac{1}{R})^2 A_{rod,vertical} = \frac{\pi\kappa\Delta L}{R}$
R is the radius of the rod. ΔL is the portion of the rod that has been engulfed.</td></tr>
<tr><td rowspan="2">Nanotile</td><td>Horizontal</td><td>$\frac{1}{2}\kappa(\frac{1}{R})^2 A_{tile,horizontal} = \frac{1}{2}\kappa\left(\frac{1}{R}\right)^2 2(L_x + w)\Delta L$
R is the membrane curvature near the edge of tile. ΔL is the portion of the tile that has been engulfed.</td></tr>
<tr><td>Vertical</td><td>$\frac{1}{2}\kappa(\frac{1}{R})^2 A_{tile,vertical} = \frac{1}{2}\kappa\left(\frac{1}{R}\right)^2 2(L_y + w)\Delta L$
R is the membrane curvature near the edge of tile. ΔL is the portion of the tile that has been engulfed.</td></tr>
</table>
